# Measuring the Evolutionary Rewiring of Biological Networks

**DOI:** 10.1371/journal.pcbi.1001050

**Published:** 2011-01-06

**Authors:** Chong Shou, Nitin Bhardwaj, Hugo Y. K. Lam, Koon-Kiu Yan, Philip M. Kim, Michael Snyder, Mark B. Gerstein

**Affiliations:** 1 Program in Computational Biology and Bioinformatics, Yale University, New Haven, Connecticut, United States of America; 2 Department of Molecular Biophysics and Biochemistry, Yale University, New Haven, Connecticut, United States of America; 3 Terrence Donnelly Center for Cellular and Biomolecular Research, Banting and Best Department of Medical Research, University of Toronto, Toronto, Ontario, Canada; 4 Department of Genetics, Stanford University, Stanford, California, United States of America; 5 Department of Computer Science, Yale University, New Haven, Connecticut, United States of America; Utrecht University, The Netherlands

## Abstract

We have accumulated a large amount of biological network data and expect even more to come. Soon, we anticipate being able to compare many different biological networks as we commonly do for molecular sequences. It has long been believed that many of these networks change, or “rewire”, at different rates. It is therefore important to develop a framework to quantify the differences between networks in a unified fashion. We developed such a formalism based on analogy to simple models of sequence evolution, and used it to conduct a systematic study of network rewiring on all the currently available biological networks. We found that, similar to sequences, biological networks show a decreased rate of change at large time divergences, because of saturation in potential substitutions. However, different types of biological networks consistently rewire at different rates. Using comparative genomics and proteomics data, we found a consistent ordering of the rewiring rates: transcription regulatory, phosphorylation regulatory, genetic interaction, miRNA regulatory, protein interaction, and metabolic pathway network, from fast to slow. This ordering was found in all comparisons we did of matched networks between organisms. To gain further intuition on network rewiring, we compared our observed rewirings with those obtained from simulation. We also investigated how readily our formalism could be mapped to other network contexts; in particular, we showed how it could be applied to analyze changes in a range of “commonplace” networks such as family trees, co-authorships and linux-kernel function dependencies.

## Author Summary

Biological networks represent various types of molecular organizations in a cell. During evolution, molecules have been shown to change at varying rates. Therefore, it is important to investigate the evolution of biological networks in terms of network rewiring. Understanding how biological networks evolve could eventually help explain the general mechanism of cellular system. In the past decade, a large amount of high-throughput experiments have helped to unravel the different types of networks in a number of species. Recent studies have provided evolutionary rate calculations on individual networks and observed different rewiring rates between them. We have chosen a systematic approach to compare rewiring rate differences among the common types of biological networks utilizing experimental data across species. Our analysis shows that regulatory networks generally evolve faster than non-regulatory collaborative networks. Our analysis also highlights future applications of the approach to address other interesting biological questions.

## Introduction

With the advent of large-scale genomic and proteomic technologies in discovering interacting and regulatory relationships in cells, many types of biological networks, though incomplete, have been constructed in various eukaryotic species [1]–[19]. The kinds of networks currently include, but are not limited to, protein interaction, genetic interaction, transcription factor-target regulatory, miRNA-target regulatory, kinase-substrate phosphorylation, and metabolic pathway. Biological networks have been used to explain differences between closely related species that share high sequence similarities [1], [2], [7]. For example, human and chimpanzee genomic sequences are found to have only 1.23% differences in SNPs and 3% in indels [20]. However, the subtle sequence divergence is hardly sufficient to explain phenotypical, behavioral and social differences between the two species. As a result, biological networks (organizations of molecules) are proposed to play a central role in speciation complementary to individual molecules [1], [2], [7]. However, it is still largely unknown how fast biological networks evolve.

Biological network research has followed the path of sequence research to some degree. In the past three decades, biological sequence research has experienced three stages: initial sequencing data generation, pairwise alignment and evolutionary rate analysis. Simple models such as the Jukes-Cantor model [21] describe evolutionary sequence divergence in terms of time. In fact, various biological sequences evolve at different rates depending upon their functional importance [22], [23]. Genomic sequence analyses in various species have helped us to learn levels of conservation among genomic regions and genes [24]–[26]. Similarly, proteomic sequence and structure analyses show that protein regions have varied evolutionary constraints [27], [28]. Analogous to sequence analysis, the development of biological network research has three similar stages: network construction by large-scale experiments and computational predictions [1]–[19], pairwise network comparison to find conserved edges as interologs or regulogs [29], [30] and building general network alignment tools [31], [32], and finally investigating levels of conservation and evolutionary change on biological networks.

One of the advantages of network study is that we can make analogies to draw intuition. For example, in commonplace social contexts, we readily observe that some “network” relationships change faster than others. Personal acquaintance networks may change in days, friendship networks and co-worker networks in months or years, while family networks change over decades. This intuition of network stability differences could be quantified and compared by the rewiring rate that reflects the nature of network relationships. Similarly, in cellular systems biological networks may rewire at various rates during evolution.

Increasingly we have seen many approaches to compare biological networks across organisms, uncovering interesting relationships of network evolution and the functional implications [7], [33]–[39]. Due to current limitations of network construction technologies and the large evolutionary distance between the species compared, the overlap between current network datasets is small. Nevertheless, the estimation of the rewiring rate in protein interaction networks is possible [33]. Various methods were used in different studies inconsistent for direct comparison, with each focused on one of the biological network types. Also, most of the studies were species specific that did not compare species with large evolutionary divergence.

Given that previous studies have set the stage, now is an opportune time to quantify network rewiring in all these comparisons in a unified way. In the past three years, more data has become available for a greater number of species covering many types of biological networks [1], [2], [4], [5], [7]. The comprehensive set of network data allows systematic comparison of rewiring rates of biological networks and drawing more robust conclusions by using a set of species pairs.

We show here the rewiring rates of several types of biological networks in eukaryotes. The approach used is consistent across network types and robust to network data quality. We observed that the rewiring rate is characteristic of the type of edge (relationship between node entities) in both biological and commonplace networks. This analysis gives an initial picture of biological network rewiring and provides intuition and useful tools for the future when more network data becomes available.

## Results

### Rewiring rate as a discriminating characteristic of networks

**Figure 1:**
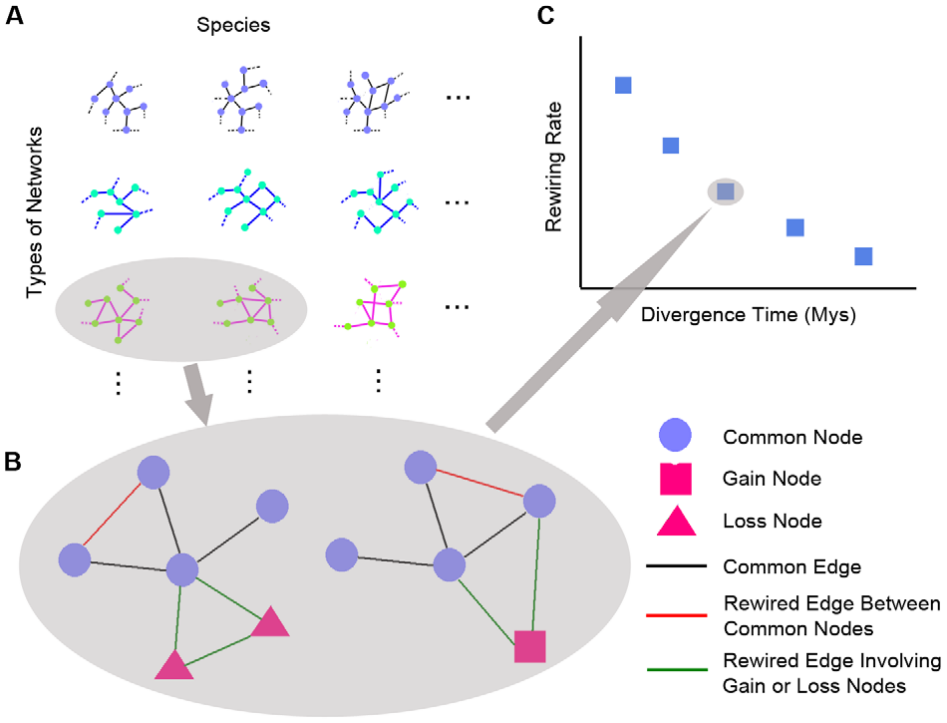
Measuring network rewiring by comparing networks of species pairs. (A) Types of biological networks with currently available data for different species are collected. Selected types of commonplace networks with multiple time-point data are also collected. (B) For each network type, we perform edge rewiring analysis for pairs of species. Three types of nodes are first identified as CNs, GNs and LNs. Four types of rewired edges are then identified and counted including gain/loss edges between CNs (red) and those involving GNs or LNs (green). Rewiring rate from comparing the networks is calculated (see Materials and Methods). (C) Rewiring rate calculated from schematic (B) corresponds to a typical result point.

To calculate the rewiring rate of biological networks, we first established node orthology between two species, and then defined edge orthology as a conserved relationship between orthologous entities across different species, which is a generalization of “interologs” in protein interaction network and “regulogs” in TF regulatory network [29], [30]. One species network is considered reference, and three sets of nodes are identified. Common nodes (CNs) are nodes present in both networks, loss nodes (LNs) only in reference network and gain nodes (GNs) only in the other compared network. Four types of rewired edges are then identified and counted including gain or loss edges between CNs, loss edges involving LNs, and gain edges involving GNs (see Figure 1). The rewiring rate was measured by the total number of rewired edges (R) between two networks normalized by the combined network size, the total number of possible edges if two networks were both “complete” (C), and divergence time (T). Total number of rewired edges (R) counts all non-conserved edges (interologs, regulogs or other type of “logs”) in two networks. The total number of possible edges (C) has five components: total possible edges of complete networks consisting of only common nodes (CNs), nodes that are only present in one of the two networks (GNs or LNs), and total possible edges between the two (between CNs and LNs, or CNs and GNs) (see Figure S1, see Materials and Methods). The measure is in essence percentage edge change of network in a given time period. We have collected data for each type of network for different species (see Table S1), and calculated rates for different time divergence species pairs (see Figure 1).

**Figure 2:**
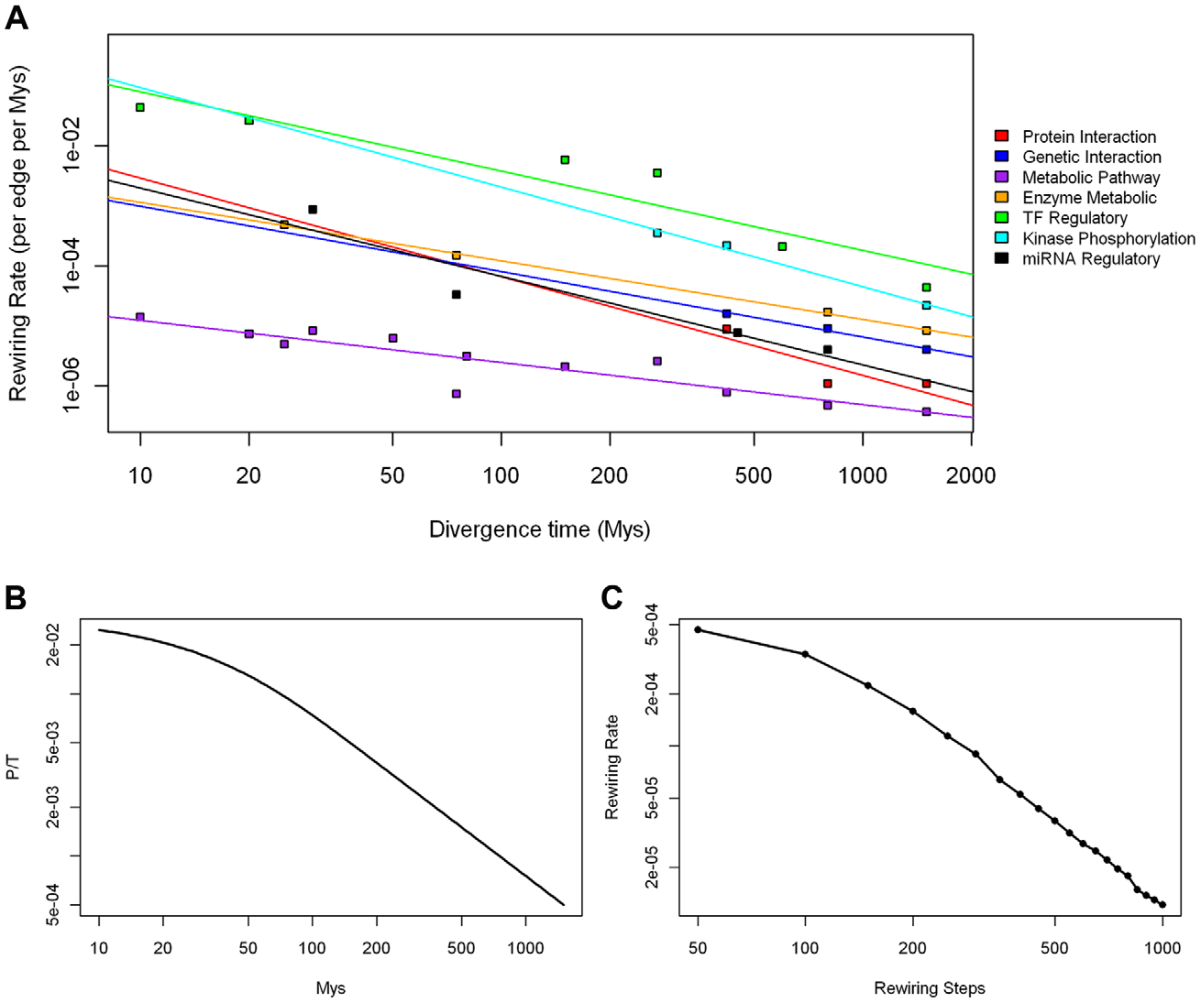
Ordering of extent of biological network rewiring. (A) Rewiring rates calculated for seven types of real biological networks (each with a different color) are shown as points on the Log-Log scale plot. Each rewiring rate corresponds to a divergence time of its two species comparison. (B) Relationship on Log-Log scale between sequence evolution rate, as number of nucleotide change per nucleotide per million years according to the Jukes-Cantor model, and divergence time. (C) Relationship on Log-Log scale between network rewiring rate from simulation and number of rewiring steps. Strong negative correlations between evolutionary rate, as percentage change per unit of time, and time are present for real networks, sequences and simulated networks.

For all types of biological networks, we observed faster rewiring rates for smaller divergence species pairs and slower rewiring rates for larger divergence species pairs, with a strong negative linear relationship between rewiring rate (per edge per Mys) and divergence time (Mys) in Log-Log scale (see Figure 2, Table S2). It was thus inappropriate to use the rewiring rate calculated from a specific species pair as a general measure for a network type. Using species pairs with different divergence times could result in large differences. However, different species pairs with similar divergence times tended to have close rewiring rates. This indicated that our rewiring rate measure was dependent upon divergence time but not on species.

We then asked the question whether the observed negative linear relationship in Log-Log scale between rate and divergence time in networks is parallel to what is seen in nucleotide sequence evolution. For sequence evolution, we use the equation 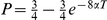 from the Jukes-Cantor model, where *P* is the percentage of sequence change and *T* is divergence time [21]. Though it is a simple model with only one parameter (*α*), Jukes-Cantor model captures the core relationship between *P* and *T*, and is sufficient in this case for comparing sequences with networks. *P/T* is the approximation of the instantaneous sequence evolutionary rate (*dP/dT*) and can be used for direct comparison with rewiring rate of networks. A negative linear relationship was observed in Log-Log scale between *P/T* and *T* (see Figure 2), and was especially strong at large divergence times.

Further, we used simulated networks to determine whether the observed relationship is specific to real biological networks. A simulation-based network rewiring model was built based on four parameters, corresponding to node changes, edge changes, and preferential attachment to rewiring networks while maintaining scale-free topology (see Materials and Methods). As a simulation of evolutionary divergence, two branches of networks were compared after the same number of rewiring steps and rewiring rates calculated (see Figure S2). The rewiring rate calculated from the simulation model also shows a negative linear relationship in Log-Log plot with number of rewiring steps (see Figure 2).

The analysis above indicated that the negative linear relationship between the rewiring rate and time in real networks could be universal and reflect underlying principles in evolution. This intuitively corresponds to the saturation of percentage change. For nucleotide sequences, as divergence becomes larger, the percentage of sequence change saturates at 0.75 according to the Jukes-Cantor model. New nucleotide changes happen on top of previous changes, which have little effect on percentage difference. Our analysis showed that the same is true for networks.

We used the fitted rates from linear models for each type at 800 Mys divergence, roughly half the time of eukaryotic history (see Table 1). The “banding” of networks on the plot into characteristic groups with order of magnitude rate differences between them indicates the robustness of the rewiring rate calculation and the actual rate difference between networks.

**Table 1:** Rewiring rate spectrum of eukaryotic biological networks.

Estimated Divergence Time (Mys)	∼25	∼75	∼270	∼800	∼1500	Fitted 800
Metabolic Pathway Network	7.4E-6	3.1E-6	4.1E-6	5.4E-7	3.7E-7	5.7E-7
Protein Interaction Network	-	-	-	1.1E-6	1.1E-6	2.2E-6
Genetic Interaction Network	-	-	-	1.3E-5	4.0E-6	8.3E-6
Metabolic Enzyme Network	4.8E-4	1.5E-4	-	1.7E-5	8.4E-6	1.6E-5
miRNA Regulatory Network	8.6E-4	3.3E-5	-	4.1E-6	-	3.1E-6
Kinase Phosphorylation Network	-	-	3.5E-4	-	2.2E-5	6.5E-5
Transcription Factor Regulatory Network	2.3E-2	-	3.5E-3	2.1E-4	4.4E-5	2.4E-4

Using estimated divergence time between species pairs (see Table S1), we calculate rewiring rates for multiple time divergence of each type of biological networks (see Materials and Methods), and show a subset of results here. ‘Fitted 800’ column is the fitted rewiring rate from linear regression at 800 Mys divergence time (see Figure 2). Network data is unavailable for rewiring rate calculation for blank cells. Rewiring rate is measured as rewiring per edge per Mys.

In fact, the above described rewiring rate is an “average” rate rather than “instantaneous” rate for networks. As the Jukes-Cantor model shows for sequences, evolutionary rate (*α*) could only be approximately measured using instantaneous rate (*dP/dT)* between closely related species (*dT* is small), where *α* is proportional to *dP/dT*. When the divergence gets large, the approximation of instantaneous rate with the average rate is poor and the relationship between *α* and *dP/dT* becomes non-linear. The logic is directly applicable to our case for networks.

Ideally, instantaneous rewiring rate should be measured using networks between closely related species. However, little network data are available for close species, which inhibits the calculation of instantaneous rewiring rates. The disadvantage of using the average rates described above is that at large evolutionary distance, network rewiring approaches saturation and is hard to compare. And the limited number of species network comparisons does not allow accurate estimations of instantaneous rates by the linear model at less than 10Mys divergence (see Table S2).

Another idea of comparing rewiring of biological networks is to use networks for a given divergence of the same species pairs. Since networks are of the same divergence, we use the percentage of edge changes among total possible changes, which is *R/C*, to measure the extent of rewiring (see Table 2). This method circumvents the disadvantages of average rewiring rate and limited species comparisons of networks, while it maintains the ability to distinguish the extent of network rewiring. For each of the 11 species comparisons listed in Table 2, biological networks are ordered according to their percentage of rewiring. We then count the number of cases where one type of biological network is observed to rewire more or less than another (see Table 3). Thus for each comparison between species (at a given level of divergence), we get an ordering of network rewiring (e.g. transcription regulatory>phosphorylation regulatory>protein interaction>metabolic pathway). We found that the ordering is consistent amongst all the 11 comparisons in this study. This result further supports the differences found in network rewiring using averaged rates.

**Table 2:** Percentage of rewired edges of eukaryotic biological networks.

Species Pair	Estimated Divergence Time (Mys)	Metabolic Pathway	Protein Interaction	Genetic Interaction	Metabolic Enzyme	miRNA Regulatory	Kinase Phosphorylation	Transcription Factor Regulatory
*S. cer, S. mik*	10	0.015%	-	-	-	-	-	43%
*S. cer, S. bay*	20	0.015%	-	-	-	-	-	46%
*H. sap, M. mul*	25	0.013%	-	-	1.2%	-	-	-
*C. ele, C. bri*	30	0.025%	-	-	-	2.6%	-	-
*H. sap, M. mus*	75	0.006%	-	-	1.1%	0.25%	-	-
*S. cer, K. lac*	150	0.032%	-	-	-	-	-	87%
*S. cer, C. alb*	270	0.11%	-	-	-	-	9.5%	95%
*S. cer, S. pom*	420	0.033%	0.37%	0.67%	-	-	9.2%	-
*D. mel, C. ele*	600	-	-	-	-	-	-	13%
*H. sap, D. mel*	800	0.033%	0.088%	1.04%	1.36%	0.32%	-	-
*H. sap, C. ele*	800	0.043%	0.088%	0.42%	1.36%	0.33%	-	-
*S. cer, D. mel*	1500	-	-	-	-	-	-	6.5%
*S. cer, H. sap*	1500	0.056%	0.17%	0.6%	1.26%	-	3.3%	-

**Table 3:** Consistency of species comparison cases of network rewiring.

	TF regulatory (T)	Kinase phosphorylation (K)	Metabolic enzyme (E)	Genetic interaction (G)	miRNA regulatory (M)	Protein interaction (I)	Metabolic pathway (P)
**T**							
**K**	T>K: 1/1						
**E**	-	K>E: 1/1					
**G**	-	K>G: 2/2	E>G: 3/3				
**M**	-	-	E>M: 3/3	G>M: 2/2			
**I**	-	K>I: 2/2	E>I: 3/3	G>I: 4/4	M>I: 2/2		
**P**	T>P: 4/4	K>P: 3/3	E>P: 5/5	G>I: 4/4	M>P: 4/4	I>P: 4/4	

The percentages of network rewiring calculated in Table 2 are compared for the extent of rewiring and summarized. ‘>’ denotes the argument of greater rewiring extent of the column type of biological network over the row type. Network types are abbreviated using capital letters in rows. Only the lower triangle of this symmetric table is filled. The ratio denotes the number of cases supporting the argument out of the total number cases compared. All arguments are supported with full consistency of species pair comparisons.

The formalism of network rewiring was also applicable to non-biological networks to get some intuition for fast or slow rewiring processes (see Table 4). Three different representative commonplace networks with very different divergences were constructed, including co-authorship networks, family trees and Linux kernel design networks (see Figure S3). The three types of non-biological networks showed differential rewiring rates in the order of magnitudes (see Table 4). Consistent with our intuition, for example, family trees have less rewiring than co-authorship networks. Contrary to popular opinion of frequent computer software updates, Linux kernel design network in fact evolves approximately one order of magnitude slower than a typical family tree (more family samples needed for statistically significant arguments). It is clear that rewiring rate could help us understand the nature of edge relationship in networks, thus can be used for direct comparisons among all kinds of biological and social networks.

**Table 4:** Rewiring rates of selected commonplace network.

	Years of Change	Rewiring Rate (per edge per year)
Linux Kernel Design Network	2	1.7E-4
Family Tree	26	9.5E-4
Lab Co-authorship Network	3	2.9E-1

Rewiring rates are calculated using the same method as for biological networks (see Materials and Methods). Notice that rewiring rate for social networks is measured in per year unit, as compared to per Mys unit in biological networks.

### Network rewiring and gene content turnover

Rewiring of biological networks consist of two sources: edge change between conserved nodes, and edge change from node gain and loss. We observed that a large fraction and in many cases the majority of network rewiring is attributed to the gain and loss of nodes (see Table S3). In fact, gene content turnover of two species contributes to the gain and loss of nodes in networks. Some studies have suggested differential gene content turnover of gene families, such as transcription factors and metabolic enzymes, in completely sequenced genomes [40]–[42]. Therefore, it is important to assess the impact of gene family evolution on the extent of their respective network rewiring.

In order to examine whether the turnover of a specific set of genes, such as kinases and TFs, have impact on their corresponding network rewiring, we examined the gene content turnover of 3 GO categories using 3 species pairs (see Table 5). The 3 GO categories (transcription factor activity, kinase activity, and metabolic process) are selected to be compared with TF-target regulatory network, kinase-substrate phosphorylation network, and metabolic enzyme network, respectively. For the 3 categories of proteins, we did not observe a clear pattern in which some categories had faster turnover than others. This suggests that differences in network rewiring across networks may not come from the gene content turnover of corresponding GO category proteins. The rewiring of networks should mostly reflect the characteristic of biological relationships rather than specific GO category molecules themselves.

**Table 5:** Gene content turnover of 3 GO categories.

		H. sapiens – M. musculus	C. elegans – C. briggsae	S. cerevisiae – K. lactis
Transcription factor	Non-conserved proteins	1864	409	19
activity	Total proteins	2785	781	235
	Content turnover	67%	52%	8%
Kinase activity	Non-conserved proteins	1977	423	7
	Total proteins	2684	817	250
	Content turnover	74%	52%	3%
Metabolic process	Non-conserved proteins	3452	606	68
	Total proteins	5227	1540	1172
	Content turnover	66%	39%	6%

Proteins in *H. sapiens*, *C. elegans* and *S. cerevisiae* from 3 GO categories are identified from annotations. In the counter species (*M. musculus*, *C. briggsae* and *K. lactis*) their orthologous counterparties are mapped. Gene content turnover for the species pair is measured as the number of non-conserved proteins over the total number of proteins in the GO category.

It is also interesting to notice that even if the fungi *S. cerevisiae* and *K. lactis* have the largest divergence of 150 Mys among three species pairs, the gene content turnover is much less than the other two pairs. This slow gene content turnover with a large species divergence further supports the role of network rewiring during evolution.

### Biological networks evolve in rates comparable to protein sequences

Cellular molecules, as nodes in biological networks, are under differentiated selection pressure, and therefore evolve at different rates. Genomic analyses from model organisms have shown the spectrum of sequence conservation among types of genomic annotations, in which protein coding exon sequences are the most conserved, intron sequences are the least conserved, and regulatory cis/trans elements are somewhere in between [43]. Proteins as the products of DNA coding sequences are generally thought to be under great constraint. Another special product from DNA sequences is ribosomal RNA, which is considered the most conserved locus in the genome [44].

We asked whether the edge rewiring rates in biological networks were in the range of node changes. Since there is no analogous concept of “total possible edges between nodes” in sequence comparisons, a naïve sequence/network identity-based method was used to measure the percentage change between two sequences/networks for consistency (see Materials and Methods). Here, only edge changes in networks are counted to compare with nucleotide change in sequences. Sequence identity is calculated as the percentage of the number of unchanged nucleotides or amino acids in global alignment per length of the alignment. Similarly, network identity is calculated as the percentage of the number of unchanged edges out of total number of edges in two networks. Then, the rate can be calculated as (1- percentage identity)/(divergence time) for both sequence and network. This equates one edge change with one nucleotide or amino acid change. We realized this might not be the best, but a default to start with.

**Figure 3:**
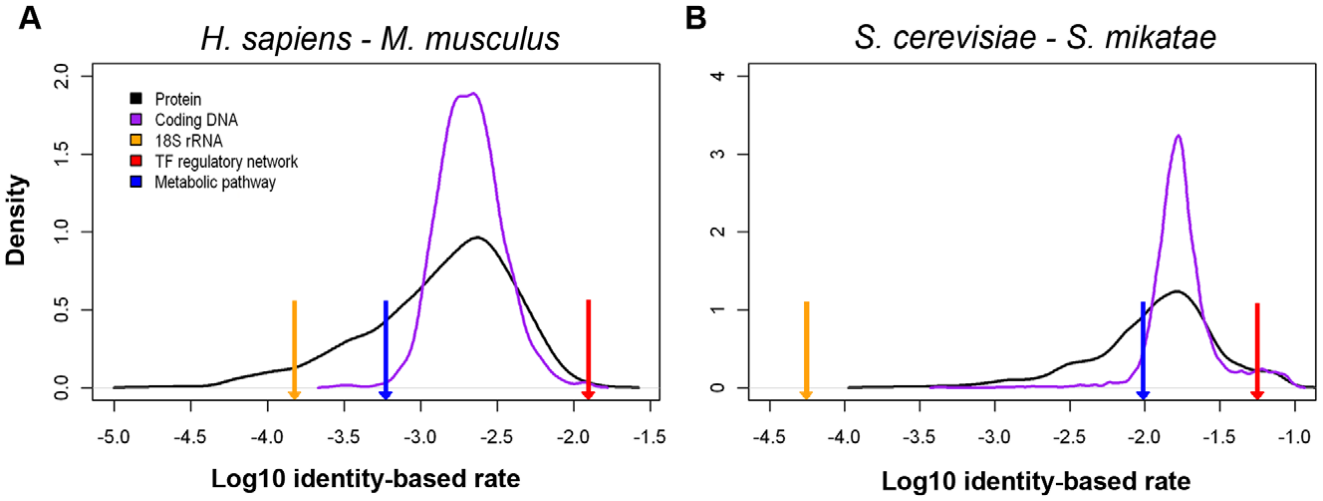
Network rewiring rates is comparable to molecular sequence change. (A) Network rewiring evolution is compared to molecular sequence evolution using *H. sapiens* and *M. musculus* data, and (B) using *S. cerevisiae* and *S. mikatae* data. Two density distributions of identity-based evolutionary rate are shown as for protein sequences (black line) and protein coding DNA sequences (purple line). 18S rRNA rate (orange arrow), transcription factor regulatory network rate (red arrow) and metabolic pathway network rate (blue arrow) are also shown for relative positions to sequence rate distributions.

Using this definition, we observed that biological networks evolve in a range comparable to that of protein sequences in both species cases (see Figure 3). Transcription factor-target regulatory networks, the fastest rewiring biological networks, were comparable to the top 0.1% and 4% of the fastest evolving protein sequences in *Homo sapiens* and *Sacchromyces cerevisiae*, respectively. The slowest rewiring metabolic pathway network was comparable to the bottom 23% and 36% of the slowest evolving protein sequences. The density distribution of protein coding DNA sequence rates had a similar peak position but a smaller standard deviation than protein sequence rates, because an amino acid change does not necessarily result from changes of all its three codon positions. Therefore the evolutionary rate distinction between protein coding sequences and biological networks became more significant: with 0.5% and 4% of sequences slower than metabolic pathway networks in human and yeast, respectively, and 0% and 4% of sequences faster than transcription factor-target regulatory networks. The 18S rRNA sequences evolved slower than all biological networks analyzed here: approximately 60% rate of the slowest rewiring metabolic pathway network in human and 1% of the rate in yeast.

### Permanent protein interactions rewire slower than transient interactions

Since rewiring rates are capable of distinguishing different network types, we attempted to use rewiring rates to study different subtypes of edges within protein interaction networks. Relating protein 3-D structures to protein interaction networks helped us to distinguish simultaneously possible (permanent) interactions from mutually exclusive (transient) interactions [45]. The difference between the two types of interactions is whether an interaction between two proteins has competition from a third potential interacting protein for the same interacting site. It has long been hypothesized that protein pairs of permanent interactions tend to co-evolve during evolution [46]. The co-evolutionary effect could help to maintain the stability of permanent interactions.

Structural interaction networks (SINs) for both human and yeast were constructed using updated and coherent datasets. Permanent and transient interactions were identified through interacting site regions in proteins and number of interacting partners for each site. Conservation of permanent and transient interactions was measured by their presence in another reference species network (see Table 6). Significant conservation distinction was observed for permanent and transient interactions in both yeast (p-value = 0.001) and human networks (p-value = 0.05) using Fisher's Exact Test. Stronger conservation of permanent protein interactions indicated that the interacting sites within two proteins were more constrained to maintain the interaction via co-evolution of interacting sites.

**Table 6:** Permanent protein interactions rewires slower than transient interactions.

Edge Type	Human Permanent	Human Transient	Yeast Permanent	Yeast Transient
Conserved	8	8	38	66
Non-Conserved	1088	2874	318	1106
Total	1096	2882	356	1172

We distinguish permanent and transient edges for protein physical interactions. Fisher's Exact Test is performed to test conservation difference between permanent and transient edges, with P-value = 0.05 for human and P-value = 0.002 for yeast. Human network edges are compared to *D. melanogaster* for conservation, and yeast *S. cerevisiae* network is compared to *S. pombe*.

### Paralogs rewire at a close pace in protein interaction networks

**Figure 4:**
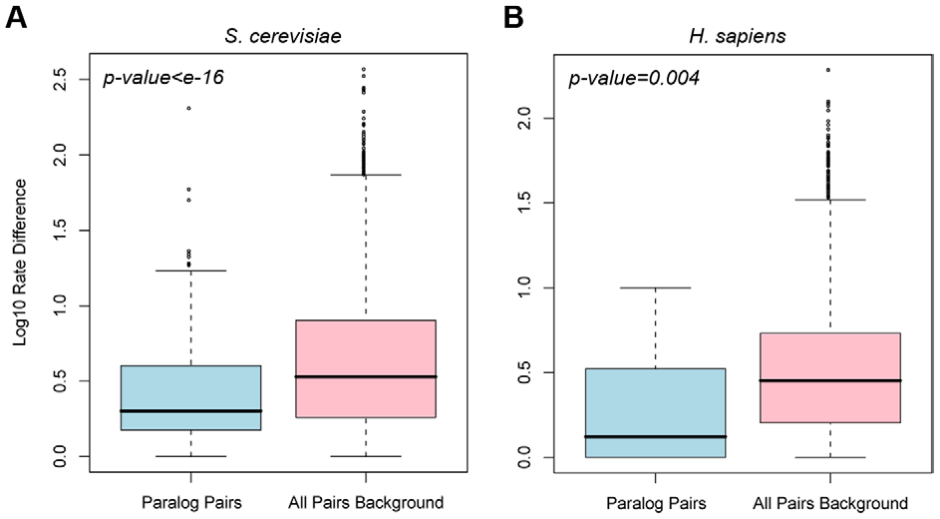
Rewiring rate difference of paralog pairs in protein interaction networks. (A) Boxplot of rewiring rate difference in yeast and (B) human protein interaction networks between paralog pairs (blue) and between all node pairs as background (pink). Paralog pairs tend to have smaller rewiring rate difference than expected.

The results above showed that the rewiring rate of network edges reflects the biological nature of edge types. It is also plausible that proteins with different characteristics might have different rewiring rates than their network partners. Here, we used protein interaction networks to investigate how protein paralogs behave during evolution in terms of changing their interacting partners. We collected all paralog pairs present in human and yeast interaction networks and calculated the rewiring rate difference between each pair (see Materials and Methods). The distribution of the rate difference was then compared with a background distribution calculated for all protein pairs in the networks (see Figure 4).

In both human and yeast networks, the paralog pairs had rate difference distribution shifted to zero compared to background (Wilcoxon test p-value<e^−15^ in yeast, p-value = 0.004 in human). The result suggested that paralog pairs tend to have a smaller rewiring rate difference, demonstrating a closer evolutionary rate of network change. In fact, as paralogs emerge from the event of gene duplication in ancestral species, they share sequence similarities [47]. Here, we showed that paralogs also shared network similarities as the network rewiring rates of paralogs were similar. After the gene duplication events which lead to their formation in ancestral species, paralogs are likely to have similar constraint on sequences and network partners due to their shorter evolutionary history than random protein pairs.

## Discussion

King and Wilson proposed [48] and Bourman et al. [1] then demonstrated that fast changing regulatory relationships in transcription factor-target networks could account for the species differences, which could hardly be explained by the highly conserved protein and DNA coding sequences. Following that study, small- and large-scale evidence has been presented to support the view that after the divergence of two species, fast change in regulatory relationships may have a critical role in speciation [2], [7]. As we have shown above, transcription factor-target regulatory networks and kinase-substrate phosphorylation networks are two major types of regulatory networks that have the fastest evolutionary changing rates among networks and protein sequences, confirming the importance of regulation in species evolution.

### Assessing network data quality to rewiring rate

Unlike sequence data that one is essentially sure of every base, network data either generated from experiments or computational predictions are currently subject to high number of false positives and false negatives. Because many distinct experimental approaches are used to generate network data, different biological networks may have varied systematic bias during their construction. It is inevitable that our results might be subject to change when new network data become available.

For each type of biological networks, we used consistent data source and method to build networks for species, which ensures the uniform definition of edges and facilitates comparison between species.

Instead of trying to build high quality networks for all biological networks in multiple species, which is difficult due to lack of gold-standard positives and negatives, we applied a general method to assess the influence of false positives and negatives to rewiring rate calculation for all biological networks. Beltrao et al. have used a sampling-based sensitivity analysis to assess the robustness of rewiring rate relative to the amount of protein interaction data used [33]. Here, we applied a similar method to six representative types of biological networks used in this study. The effects of false negatives and false positives are simulated by random sampling. That is, we randomly add and remove a fraction of edges of the two compared real networks, forming simulated “corrected” networks, and then calculate rewiring rates. A series of disruption fractions of random edges are used to simulate false positive and negative rates from low to high (see Figure S5, see Materials and Methods).

Rewiring rates of most of the biological networks are robust to network size change and disruption, especially when the disruption fraction is lower than 50%. However, the rates of metabolic pathway networks have shown clear deviations at large disruption levels. The observed one order of magnitude difference between metabolic pathway networks and protein interaction networks (10^−5^ for protein interaction network, 10^−6^ for metabolic pathway) disappears at approximately 70% disruption level. We conclude from these results that the network rewiring rate is only slightly affected by network size, and is especially robust at sampling levels above 50%. The results of this study should still hold when new network data arrives.

We also investigated the potential size effect of fungi TF-target regulatory networks used in our study. These networks were constructed using binding sites from ChIP-chip experiments of one or two TFs, which results in relatively small networks. Besides the simulated disruption described previously on these small networks, edges were added to the *S. cerevisiae* network from another ChIP-chip study between the existence nodes to generate a larger network [49]. The same disruption analysis was performed on the larger network. Rewiring rates calculated from the larger network decreased about half order of magnitude than from the original small network (see Figure S5). This is largely due to the increase of total possible edge changes in our calculation. As a result, the current subnetwork of TF-target regulatory network might lead to a bias of faster rewiring rate.

A comprehensive simulation analysis was further performed to assess the effects of both network size and network quality (see Materials and Methods). Two simulated scale-free networks were constructed with some common edges, and sub-samples of both networks were taken for comparison. Random rewiring of both sub-network were performed to mimic false positives and negatives. Percentage of edge change (R/C) was calculated for each sub-sampling fraction. As the size of the compared sub-networks decreases, percentage of rewiring increases (see Table S4). The upward bias of percentage of rewiring is approximately one order of magnitude corresponding to 1% sub-sampling fraction. Because the fungi TF regulatory network used in this study is approximately 20–100 times smaller than the complete networks estimated by the number of edges and the number of TFs [49]. We thus estimated that the true rate of fungi TF regulatory network could be half to one order of magnitude slower than we calculated. Considering the above estimation of network size effect on rewiring measurement, fungi TF regulatory network should still rewire faster than or in a similar pace as kinase phosphorylation network, and much faster than other types of biological networks (see Table 1).

miRNA regulatory networks were constructed using a consistent miRNA target prediction method [50]. In the current stage of miRNA research, most miRNAs are found or predicted using sequence conservation, and regulatory relationship is predicted mainly by searching for complementary sequence in 3′ UTRs [9]–[11]. Therefore, the turnover of miRNAs is small with lack of species-specific miRNAs and their corresponding targets. For example, a total of 459 conserved miRNAs are present in the networks comparing human and mouse. However, only 18 and 9 miRNAs are human-specific and mouse-specific, respectively. The mere gene content turnover of only 6% for miRNAs is much less than 67% and 74% for TFs and kinases (see Table 5). This ascertainment bias could result in under-estimation of rewiring rates.

To estimate the effect of novel miRNAs to our rewiring measurements, we randomly added a series numbers of hypothetical novel miRNAs to actual human and mouse miRNA regulatory networks. The targets of those hypothetical miRNAs are also randomly selected with degree distribution maintained. Rewiring rates calculated from these simulations showed that discovering potential species-specific miRNAs could result in an increase of rewiring rate (see Table S5). With the advance of miRNA research from novel miRNA discovery to better target prediction methods, it is possible that the current rewiring rates of miRNA regulatory networks will be adjusted higher.

### Rewiring rate calculation and sensitivity analysis

For all types of biological networks and simulated networks we observe a negative linear relationship between rewiring rate and divergence time (see Figure 2). Generally speaking, the average rewiring rate calculated comparing distant species networks tends to be smaller than the instantaneous rate comparing close species networks. For networks from two distant species, overlap of their nodes becomes smaller due to loss of conservation. As a result, the total number of possible edges *C* increases and rewiring rate decreases correspondingly. In conclusion, a larger difference between node sets of two distant species networks might be the main reason for this bias.

The major effect of node gain and loss on rewiring rate was further confirmed by a sensitivity analysis based on network rewiring simulation model (see Materials and Methods). Each of four independent parameters in our model was tested for its relative importance to model output—rewiring rate. Not surprisingly, we found that some parameters are more significant to the model than others. Removal of node has the strongest effect (negative linear) on rewiring rate, because rewired edges associated with a node are removed along with the node, which decreases the total number of rewired edges. Adding node also has some effect (positive linear) on rewiring rate, because of the increased number of total rewired edges associated with the node. Nevertheless, removing and adding edges have only small effects on rewiring rate (see Figure S4). It is reasonable that removing and adding nodes has a major influence on rewiring rate as it affects *all* edges associated with nodes rather than individual edges.

It is also possible that there are “cores” for each type of networks that slow down the rewiring process when it approaches the cores. The cores are partial networks that are the most constrained and conserved during evolution, possibly reflecting their functional importance. Therefore, network types with a smaller ratio of rewiring rate changes and divergence time (flat lines) might have larger cores, because of greater resistance to rewire the cores; while network types with a larger ratio (steep lines) might have smaller cores (see Figure 2).

### Collaborative networks and regulatory networks

Biological networks are characterized by their functional relationships: protein binding, expression regulation, phosphorylation, etc. We introduce another way to categorize biological networks into collaborative and regulatory networks by the reversibility of edges to help understand rewiring rate distinction among network types. Collaborative networks are the biological networks with reversible edges—either the edges are undirected or directed but reversible. By reversibility we mean that a reversed edge is biologically possible between a pair of nodes. Regulatory networks have irreversible edges: a reversed edge may not be biologically possible. By this definition, transcription factor-target regulatory networks, miRNA-target regulatory networks, and kinase-substrate phosphorylation networks fall into the regulatory network group; and protein interaction networks, genetic interaction networks, and metabolic networks fall into the collaborative network group.

Our network rewiring analysis shows that in general, regulatory networks tend to rewire faster than collaborative networks (see Table 1). Two of the regulatory networks, transcription factor-target regulatory networks and kinase-substrate phosphorylation networks, are the fastest rewiring biological networks in this study. Transcriptional regulation of gene expression by transcription factors is carried out by transcription factor binding to the transcription start site commonly upstream of a gene. Recognition of a binding site is often specific to a sequence pattern buried in the site [51]. Post-translational modification of protein substrate by kinases also involves recognition of sequence patterns in substrate's phosphorylation site [52]. Sequence pattern matching as a major factor in establishing regulatory relationships could be an important reason of fast rewiring. A single nucleotide/amino acid change in the target's binding-recognition sites, could lead to a “digital” recognition site change. Besides, a number of studies have showed that both transposable element insertion and genomic rearrangement led to considerable indel changes at transcription factor binding sites [53]–[58]. The digital and indel changes in binding-recognition sites greatly contribute to the large turnover of transcription factor-target regulatory network.

Collaborative networks show slower rewiring rates than regulatory networks. Contrary to “digital” or “indel” changes in regulatory networks, changes tend to be “structurally continuous” in collaborative networks. Here, we generally refer to the globular interactions which are the majority in physical interaction networks. On the other hand, the general collaborative physical interaction network in this study still includes interactions mediated by kinases and domains such as SH3 which are in fact regulatory relationships. In fact, protein functions gradually change as sequence changes, and most proteins do not change their functions radically with their sequences conserved. As a natural implication of the sequence-function paradigm, it is not surprising that collaborative protein networks rewire as protein sequences evolve. In this study we include two representations of metabolic networks. Metabolic enzyme networks are constructed using enzymes as nodes and edges connect two nodes if the product of one serves as the substrate of the other. The rewiring rates of metabolic enzyme networks are similar to other collaborative networks (see Table 2). On the other hand, metabolic pathway networks that are constructed using chemical compounds as nodes and reactions as edges rewire the slowest. For example, the biosynthesis metabolic pathway of acetyl-CoA from pyruvate is identical in human and yeast, but the corresponding metabolic enzyme network rewires (see Figure S6). In fact, metabolic reactions process chemical compounds into energy and nutrition, and are mostly essential for living. Our results suggest that the essentiality is partly reflected in the slower rewiring rate of metabolic pathway networks than that of other types of biological networks and protein sequences. Based on these results, we think that enzymes for reactions are less constrained to change while the underlying reactions remain highly conserved.

### Network rewiring as an important aspect of cellular system evolution

We now know that there are two layers of cellular evolution, individual molecules and organizations of molecules. Therefore, it is our ultimate goal to understand how individual molecule changes affect cells and their organization and collaboration.

**Figure 5:**
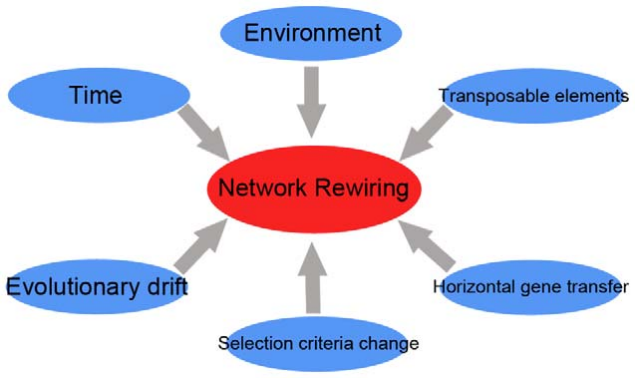
Factors shaping network rewiring.

Some factors may also influence and shape the landscape of biological networks (see Figure 5). It has been shown that external environment can influence the conservation of regulatory relationship and network motifs in prokaryotic transcription factor-target networks [59], [60]. Relationships tend to be conserved in organisms living in similar environmental niches, despite large evolutionary distance. Whole-genome duplication events rapidly reorganized transcription regulatory networks through the survived duplicates and their functional divergence afterwards [61]–[65]. And the regulatory networks, in a feedback way, could affect the survival of duplicated genes [66].

This study attempts to systematically investigate the evolutionary rate of all known types of biological networks in terms of rewiring. According to our results, it is possible that small changes of molecular sequences lead to large network re-organizations and this augmentation effect makes small molecular changes more detectable by natural selection. This is especially true for regulatory networks with the greatest augmentation effects caused by minor changes of regulators. If the above assumptions are true, network rewiring should be an essential tool to understand the differences between closely related species such as human and chimpanzee, because their molecular sequences are nearly identical. More importantly, intra-species network rewiring variations will help at an individual level beyond SNPs and structural variations.

### Future directions of network rewiring analysis

In the future, we foresee additional calculations and analyses that could be performed when accurate and more complete network data becomes available for more species. Analogous to sequence analysis, we can build species trees comparing biological networks and infer branch lengths using rewiring rates. From this study, we know that types of biological networks and molecular sequences evolve at different rates, but it is still unclear whether network rewiring “speeds up” in some species and “slows down” in others. We can use benchmark rates and develop comparative ratios to measure this. This is actually quite similar to using dN/dS ratio (non-synonymous changes versus synonymous changes) to measure selection pressure on coding sequences. Building the tree is important to understanding biological system evolution compared to traditional molecular evolution.

Network hubs and bottlenecks are of general interest in biological research due to their topological importance. Both hub and bottleneck proteins in human and yeast protein interaction networks tend to rewire their edges faster than non-hub non-bottleneck proteins (see Figure S7). One reason for this is that hubs with large degrees tend to have more rewired edges, and therefore faster rewiring rates. Further detailed analysis is needed to understand the rewiring rates of bottleneck proteins.

It is also interesting to look for rewiring “hotspots” and “coldspots” within biological networks. Subnetworks and motifs that are enriched in fast or slow rewiring edges may have biological function implications. Immune response, transport and localization associated genes in human protein interaction networks have been found to change interacting partners relatively quickly [33]. The analysis could also be applied to other types of biological networks.

Further network rewiring analysis will possibly investigate factors affecting network rewiring (see Figure 5). These efforts will greatly increase our understanding of cellular system evolution, intra-species variation, and speciation.

## Materials and Methods

### Datasets of networks, sequences and homologs

For different types of biological networks, we gathered data from multiple sources. Binary protein physical interaction networks and genetic interaction networks were extracted from BioGRID database v2.0.55 (http://thebiogrid.org/) for 5 species: *H. sapiens*, *C. elegans*, *D. melanogaster*, *S. pombe* and *S. cerevisiae* [67]. Metabolic pathway networks of compound reactions were obtained from KEGG database (http://www.genome.jp/kegg/) for 16 species: *H. sapiens*, *M. mulatta*, *M. musculus*, *C. elegans*, *C. briggsae*, *D. melanogaster*, *D. pseudoobscura*, *S. pombe*, *D. hansenii*, *C. albicans*, *K. lactis*, *C. glabrata*, *S. bayanus*, *S. mikatae*, *S. paradoxus* and *S. cerevisiae* [68]. Metabolic enzyme networks were constructed from the pathway networks for 7 species: *H. sapiens*, *M. mulatta*, *M. musculus*, *C. elegans*, *D. melanogaster*, *D. hansenii*, and *S. cerevisiae*, by establishing directed edges from upstream reaction enzymes to downstream reaction enzymes. miRNA-target regulatory networks were constructed from miRBase (http://www.mirbase.org/) predictions with edges pointing from miRNAs to target genes in 5 species: *H. sapiens*, *M. musculus*, *D. rerio*, *C. elegans* and *D. melanogaster* [50]. Transcription factor-target regulatory networks were extracted from various sources: *S. cerevisiae*, *C. elegans* and *D. melanogaster* networks from large-scale ChIP-Chip and ChIP-Seq experiments [3]–[5], *C. albicans*, *K. lactis*, *S. bayanus*, *S. mikatae* networks from recent small-scale experiments [1], [2]. Kinase-substrate phosphorylation network for *S. cerevisiae* was obtained from large-scale protein chip experiments [6]. Phosphorylation networks of yeast species *S. cerevisiae*, *C. albicans* and *S. pombe* were constructed in two steps. We first obtained phosphorylation sites identified by MassSpec [7], and also obtained kinase binding specificity data from kinase binding specificity experiments [69]; then used MOTIPS analysis pipeline to identify responsible kinases for each phosphorylation site by matching position weight matrices (PWMs) [70]. Structural Interaction Networks (SINs) for *H. sapiens* and *S. cerevisiae* were constructed in a similar way as the first version of yeast SIN [43], using protein domain interaction data from iPfam database Release 20.0 (http://ipfam.sanger.ac.uk/) [71].

For social co-authorship network, we parsed the co-author lists of 2009 Nobel Prize Winner Thomas A. Steitz's 2009 and 2006 publications from PubMed (http://www.ncbi.nlm.nih.gov/pubmed/) [69], and constructed co-authorship networks for Dr. Steitz. For social family tree network, we obtained data from a typical family with its trees in 1983 and 2009 (see Figure S3). Edges in family trees stand for either marriage or child/parent relationship. Linux kernel design networks are obtained for 3 versions, v2.6.4, v2.6.15 and v2.6.27. From v2.6.4 to v2.6.15 and from v2.6.15 to v2.6.24, the time separations are around 2 years and 2.5 years, respectively [72]. One edge in Linux kernel design networks represents one function calling or using another function.

Protein sequences and protein coding DNA sequences for *H. sapiens*, *M. musculus* and *S. cerevisiae* were downloaded from BioMart database (http://www.biomart.org/) [73], and from SGD (http://www.yeastgenome.org/) for *S. mikatae*. 18S ribosome RNA sequences for all 4 species were extracted from Entrez database (http://www.ncbi.nlm.nih.gov/Entrez/) [74]. Orthologous sequences in *H. sapiens*-*M. musculus* and *S. cerevisiae*-*S. mikatae* pairs were then aligned using MUSCLE software v4.0 (http://www.drive5.com/muscle/) [75] for calculations of sequence identity.

Sequence orthology for non-fungi species pairs used in this study was downloaded from InParanoid database v7.0 (http://inparanoid.sbc.su.se/cgi-bin/index.cgi) [76]. Orthology for fungi species pairs was obtained from Fungal Orthogroups Repository v1.1 (http://www.broadinstitute.org/regev/orthogroups/) [77]. Paralog pairs in *H. sapiens* and *S. cerevisiae* were extracted from HomoloGene database Release 64 (http://www.ncbi.nlm.nih.gov/homologene) [74].

### Calculating network rewiring rates

We used a consistent method to calculate rewiring rates comparing two networks for all network types. First, orthology relationships between nodes from the same network type in two species were established. Second, three sets of nodes were distinguished. Common Node (CN) set includes nodes having orthologous counterparts present in both networks. Loss Node (LN) set includes nodes present in the reference network but absent of orthologous counterparts in the compared network. And Gain Node (GN) set includes nodes present in the compared network but not having orthologous counterparts present in the reference network. Third, we counted the total number of rewired edges (R) between two networks. Rewired edges between two networks were defined as the union of edges between pairs of CNs that only present in one network and all edges involving LNs and GNs. Fourth, we counted the total number of possible edges (C) in the two networks. This was basically the number of non-redundant edges if two networks are both fully connected. Finally, the following equation was used to calculate the rewiring rate for a pair of networks:
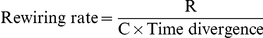
The time divergence is either estimated evolutionary divergence time (in Mys) between two species in biological networks or passed period of time (in years, and then coerced to Mys) in commonplace networks (see Table S1). Thus, the rewiring rate was measured as the number of rewired edges per edge per Mys. It can be interpreted as the averaged fraction of rewired edges among all possible edges in a period of one million years.

However, total number of possible edges was calculated differently among network types. Calculation for collaborative networks, including social networks, is simpler because their edges are reversible (see Figure S1):
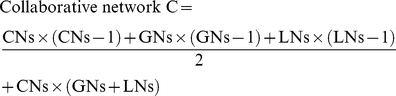
Note that here we did not allow self interactions and only allowed one edge between two nodes. For metabolic networks that allow two reciprocal edges between two nodes (for directional reactions), we just multiplied the above calculated result by 2. For regulatory networks involving irreversible edges, we further separated nodes into regulators (Regs) and targets (Tars) and only allowed edges from Regs to Tars, but not from Tars back to Regs. In addition, regulators in transcription factor-target regulatory network and kinase-substrate phosphorylation network could themselves be targets of other regulators, but not in miRNA-target regulatory network. Considering all these factors (see Figure S1),
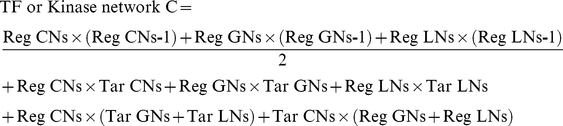
and
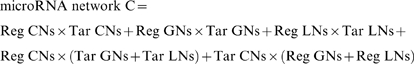



### Correlation between rewiring rate and divergence time

Rewiring rates and their corresponding estimated divergence times were plotted on Log-Log scale and then fitted with linear regression model. Using species pairs with divergence time of *t* Mys, the rewiring rates, *r*, was then regressed for each type of biological networks (see Table S2).

### Calculating evolutionary rates in network and sequence comparisons

The rewiring rate calculation described above was not directly comparable to sequence evolution rate calculation, as there is no equivalent to the ‘total number of possible edges’ as in networks. Therefore, we used identity-based evolutionary rate measures instead to compare networks and sequences as:
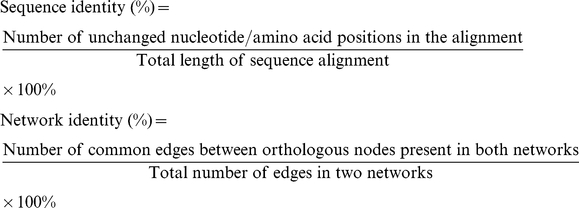
The evolutionary rate calculated based on identity was:
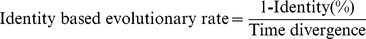



### Calculating rewiring rate difference for paralog pairs in protein interaction networks

Rewiring rates for all individual nodes were calculated for *H. sapiens* and *S. cerevisiae* protein interaction networks by comparing them to *D. melanogaster* and *S. pombe* networks, respectively. Number of rewired edges for each node was counted as the number of gained or lost edges involving this node. This number was then divided by network size and by divergence time to get rewiring rate for a node. Network size is difference for CNs, GNs and LNs. For CNs, network size is the sum of the number of CNs, GNs and LNs from the two networks; for GNs, network size is the sum of CNs and GNs; and for LNs, network size is the sum of CNs and LNs. Rewiring rate difference was then calculated for all node pairs including all paralog pairs.

### Simulation model of network rewiring

The model had four parameters: probabilities of adding a node (adding one edge with that node using preferentially attachment), removing a node (randomly for all existing nodes and all edges with that node), adding an edge (using preferentially attachment) and removing an edge (randomly for all existing edges). Preferential attachment mechanism maintains the scale-free topology of networks. To begin with, a small scale-free network was used as a seed to the model. For each rewiring step, nodes and edges were added/removed according to the probability parameters, and the resulting network was recorded for the next step.

For the relationship analysis of rewiring rate and rewiring steps, two independent rewiring branches were simulated with each 1000 steps (see Figure S2). The networks from the two branches were compared after every 50 steps and rewiring rate was calculated.

For parameter sensitivity analysis, 200 parameter-set samples were generated, with the four probability parameters randomly generated from a uniform distribution on the interval [0,1]. The same seed network was used for all 200 simulations using the 200 random parameter-sets. All simulations were stopped after 100 steps and rewiring rate was calculated corresponding to each of the 200 parameter-sets.

### Simulation of network size, false positive and false negative rates

Two simulated scale-free networks were built with some common edges for comparison. The pair of networks were sub-sampled of their edges to a series of fractions, from 95% to 1%. To assess the amount of false positives and false negatives in network data to rewiring rate calculation, we further perturbed the compared network pair (either real biological networks or simulated networks) by randomly adding and removing edges on both networks. Edges were added using preferential attachment. A series of perturbation percentages were used to simulate levels of false positive and negative rates.

## Supporting Information

Figure S1 Schematic of total number of possible edges calculation in rewiring rate. (A) For collaborative networks including protein interaction network, genetic interaction network and metabolic networks. Solid circles represent sets of nodes, as common nodes (CN), gain nodes (GN) and loss nodes (LN); dashed circles conceptually represent individual networks. Lines represent complete number of undirected edges between node sets, with each corresponding to a term in total number of possible edges summation. (B) For TF target regulatory network and kinase-substrate phosphorylation network. TFs or kinases are shown as regulators (Reg), and TF target genes or substrates as targets (Tar). Arrows represent complete number of directed edges between node sets. (C) For miRNA target regulatory network. miRNAs are shown as regulators (Reg) and their target genes as targets (Tar).
(0.24 MB TIF)

Figure S2 Simulation of network rewiring and rewiring rate calculation. Simulation of network rewiring started from a seed network, and had two independent branches of simulation. Each branch had 1000 rewiring steps, and snapshots of rewired networks were taken every 50 steps. For each rewiring step, the starting network was rewired to generate the next network according to the same parameter set. Rewiring rate was calculated comparing two independently rewired networks from two branches with the same number of steps, e.g. 50, 100, 150, 200 and all the way to 1000. This simulated rewiring rate calculation comparing species of different time divergence.
(0.05 MB TIF)

Figure S3 Visualization of types of social networks. (A) A typical family tree in 1983 (red edges) and in 2009 (blue edges), with unchanged nodes aligned. (B) Dr. Steitz Lab co-authorship network in 2006 (red edges) and in 2009 (blue edges).
(0.23 MB TIF)

Figure S4 Sensitivity analysis of four network rewiring parameters to rewiring rate. Four parameters in our rewiring simulation model - probabilities of adding a node, removing a node, adding an edge and removing an edge, are analyzed for their importance to calculated network rewiring rate. Removing node probability has the greatest negative effect on rewiring rate calculation.
(0.10 MB TIF)

Figure S5 Sensitivity analysis of false positive and false negative rates to rewiring rate. We sampled biological networks in order to test the false positive and false negative rates to rewiring rate calculation. Six biological networks are included here: protein interaction network, genetic interaction network, miRNA-target regulatory network, kinase-substrate phosphorylation network, metabolic pathway network (*S. cerevisiae* compared to *S. pombe*) and transcription factor target regulatory network (*S. cerevisiae* compared to *S. bayanus*). For each type of network, we randomly delete and add edges from the original network as a simulation of false positives and false negatives, with each a series of percentage disruptions. For transcription factor target regulatory network, we also tested rewiring rate sensitivity to network size by using a larger original network for *S. cerevisiae*.
(0.11 MB TIF)

Figure S6 Example rewiring of metabolic pathway network and metabolic enzyme network. (A) The biosynthesis pathway of acetyl-CoA from pyruvate showing metabolites (circles) and reactions (arrows). The pathway is identical in human and yeast. (B) The corresponding metabolic enzyme networks in yeast and human showing enzymes (rectangles) and product-substrate relationships (arrows). Each enzyme corresponds to a reaction in (A). Purple rectangles represent orthologous enzymes from two species, while green rectangles represent non-orthologous enzymes. The dashed circle shows one yeast enzyme coded by YER178W catalyzes two consecutive reactions, but different enzymes catalyze each reaction in human.
(0.19 MB TIF)

Figure S7 Rewiring rate of hubs and bottlenecks in protein interaction networks. Rewiring rates are calculated for all proteins in (A) human and (B) yeast protein interaction networks. Hubs are defined as top 20% proteins ranked by their degree, and bottlenecks as top 20% ranked by betweenness. Proteins are grouped into 4 categories: Bottleneck hubs (BH), Non-bottleneck hubs (NB-H), Non-hub bottlenecks (NH-B) and Non-hub non-bottlenecks (NH-NB). Either hubs or bottlenecks are found to have faster rewiring rates than NH-NBs (Wilcoxon p-val<e-15).
(0.09 MB TIF)

Table S1 Estimated divergence times between species pairs. All species pairs used in this study for calculating rewiring rates comparing species networks are listed with estimated divergence time in evolution. The types of networks used for each of these species pairs are also listed.
(0.04 MB DOC)

Table S2 Linear regression models of biological network rewiring rate and divergence time. For each type of biological network, rewiring rates (r) from different species pairs are regressed with divergence time (t), both in Log scale. Pearson correlation coefficient is also calculated.
(0.04 MB DOC)

Table S3 Detailed rewiring rates for networks and species pairs. Detailed information of rewiring rate results for all networks and species-pairs studied. Numbers of common nodes, gain nodes and loss nodes are provided. Four types of rewired edges (gain edge between common nodes, loss edge between common nodes, gain edge involving gain/loss nodes, loss edge involving gain/loss nodes) are also distinguished for separate rewiring rates. Note for biological networks, rewiring rates are measured by per edge per Mys, while for commonplace networks by per edge per year.
(0.17 MB DOC)

Table S4 Simulation of network size, false positives, and false negatives to rewiring rate. Based on two simulated scale-free networks, sub-networks are sampled to mimic the fact that data of many biological networks used in this study are not complete, such as the fungi TF regulatory networks. Extra random rewiring by adding and removing edges and nodes is performed to mimic the false positives and negatives in the current network data. Percentage of network rewiring is then calculated to assess the effects of those perturbations.
(0.05 MB DOC)

Table S5 Simulation analysis of the effect of novel miRNAs to miRNA regulatory network. Based on current miRNA regulatory networks for human and mouse, simulated novel miRNAs are added to both networks with their target randomly sampled, while maintaining the power-law distribution of target number distribution. Statistics are calculated comparing the simulated networks.
(0.04 MB DOC)
