# Supplementary figures and images for: Measuring the Evolutionary Rewiring of Biological Networks
Source: PLoS Comput Biol. 2011 Jan 6;7(1):e1001050. doi: 10.1371/journal.pcbi.1001050 (PMC3017101; doi:10.1371/journal.pcbi.1001050)

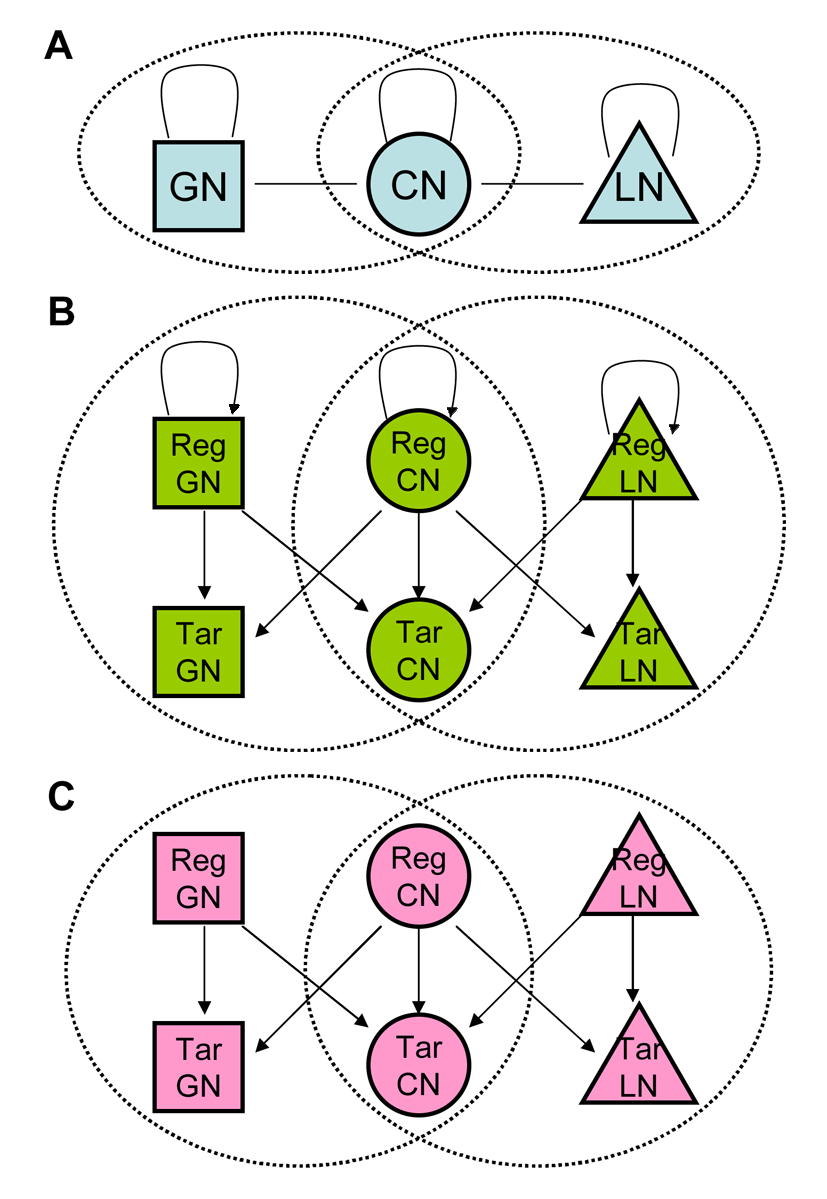

Supplement: Figure S1 — Schematic of total number of possible edges calculation in rewiring rate. (A) For collaborative networks including protein interaction network, genetic interaction network and metabolic networks. Solid circles represent sets of nodes, as common nodes (CN), gain nodes (GN) and loss nodes (LN); dashed circles conceptually represent individual networks. Lines represent complete number of undirected edges between node sets, with each corresponding to a term in total number of possible edges summation. (B) For TF target regulatory network and kinase-substrate phosphorylation network. TFs or kinases are shown as regulators (Reg), and TF target genes or substrates as targets (Tar). Arrows represent complete number of directed edges between node sets. (C) For miRNA target regulatory network. miRNAs are shown as regulators (Reg) and their target genes as targets (Tar). (0.24 MB TIF) [file pcbi.1001050.s001.tif]

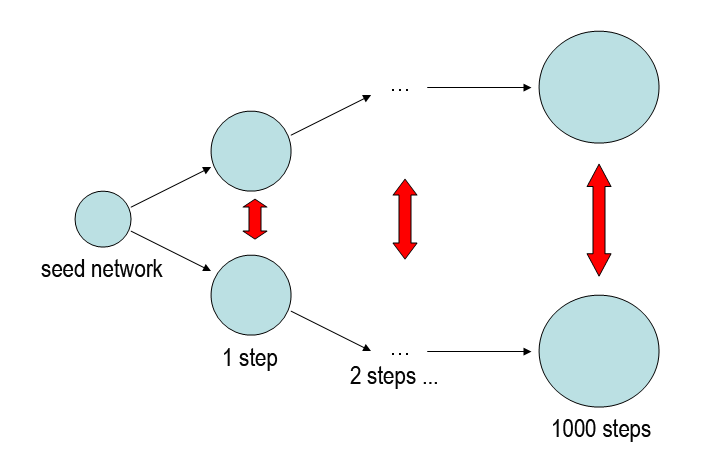

Supplement: Figure S2 — Simulation of network rewiring and rewiring rate calculation. Simulation of network rewiring started from a seed network, and had two independent branches of simulation. Each branch had 1000 rewiring steps, and snapshots of rewired networks were taken every 50 steps. For each rewiring step, the starting network was rewired to generate the next network according to the same parameter set. Rewiring rate was calculated comparing two independently rewired networks from two branches with the same number of steps, e.g. 50, 100, 150, 200 and all the way to 1000. This simulated rewiring rate calculation comparing species of different time divergence. (0.05 MB TIF) [file pcbi.1001050.s002.tif]

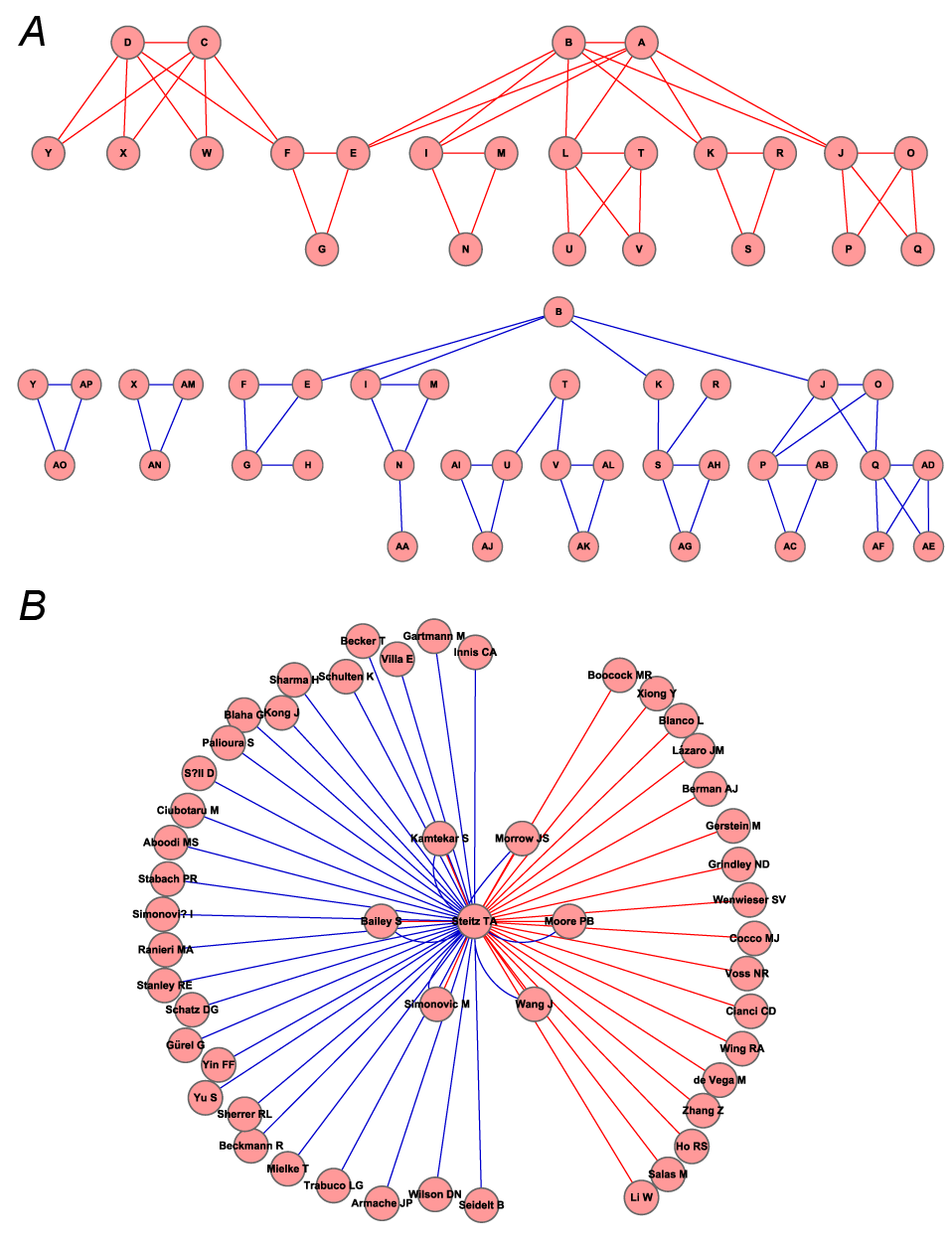

Supplement: Figure S3 — Visualization of types of social networks. (A) A typical family tree in 1983 (red edges) and in 2009 (blue edges), with unchanged nodes aligned. (B) Dr. Steitz Lab co-authorship network in 2006 (red edges) and in 2009 (blue edges). (0.23 MB TIF) [file pcbi.1001050.s003.tif]

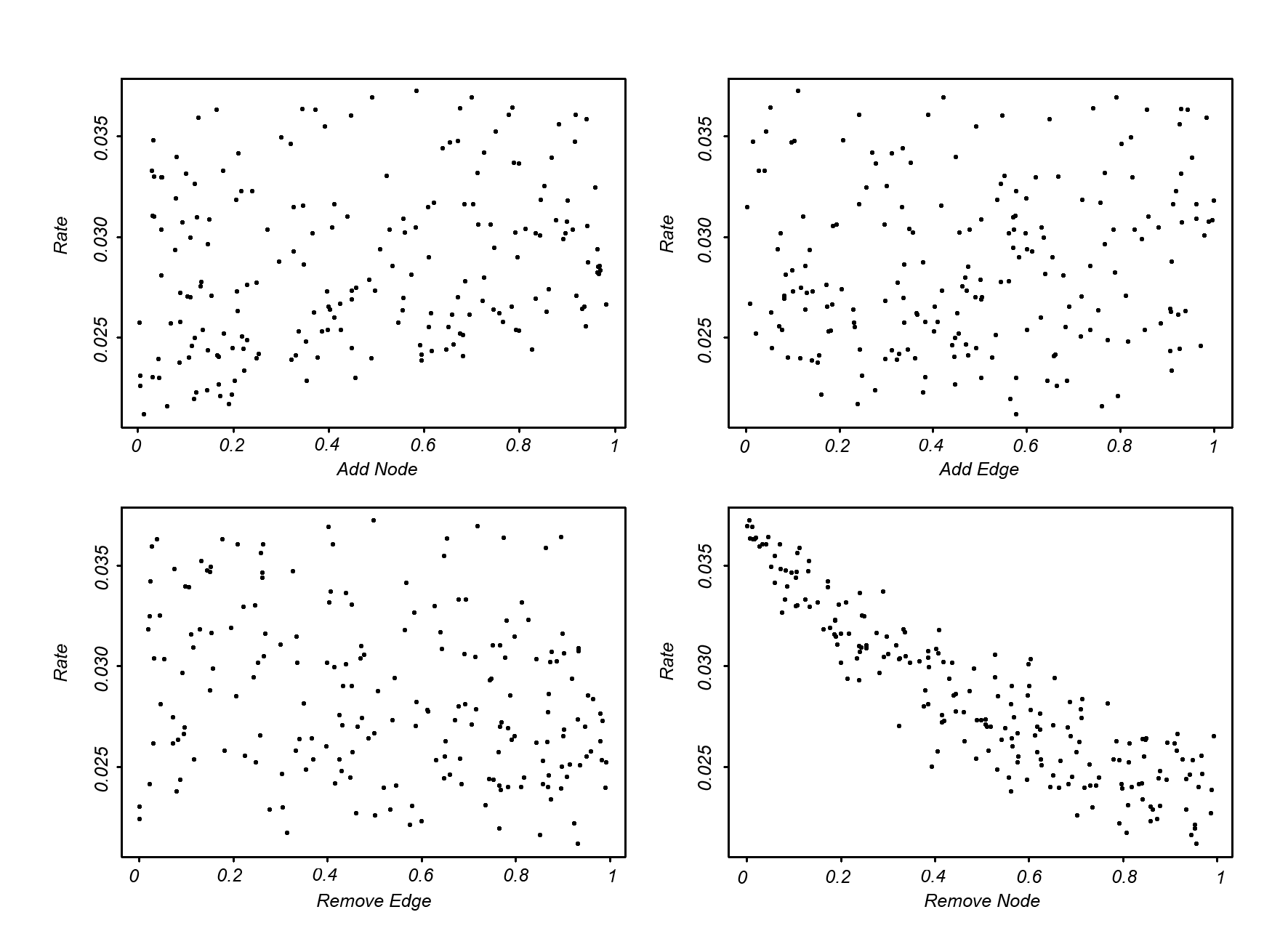

Supplement: Figure S4 — Sensitivity analysis of four network rewiring parameters to rewiring rate. Four parameters in our rewiring simulation model - probabilities of adding a node, removing a node, adding an edge and removing an edge, are analyzed for their importance to calculated network rewiring rate. Removing node probability has the greatest negative effect on rewiring rate calculation. (0.10 MB TIF) [file pcbi.1001050.s004.tif]

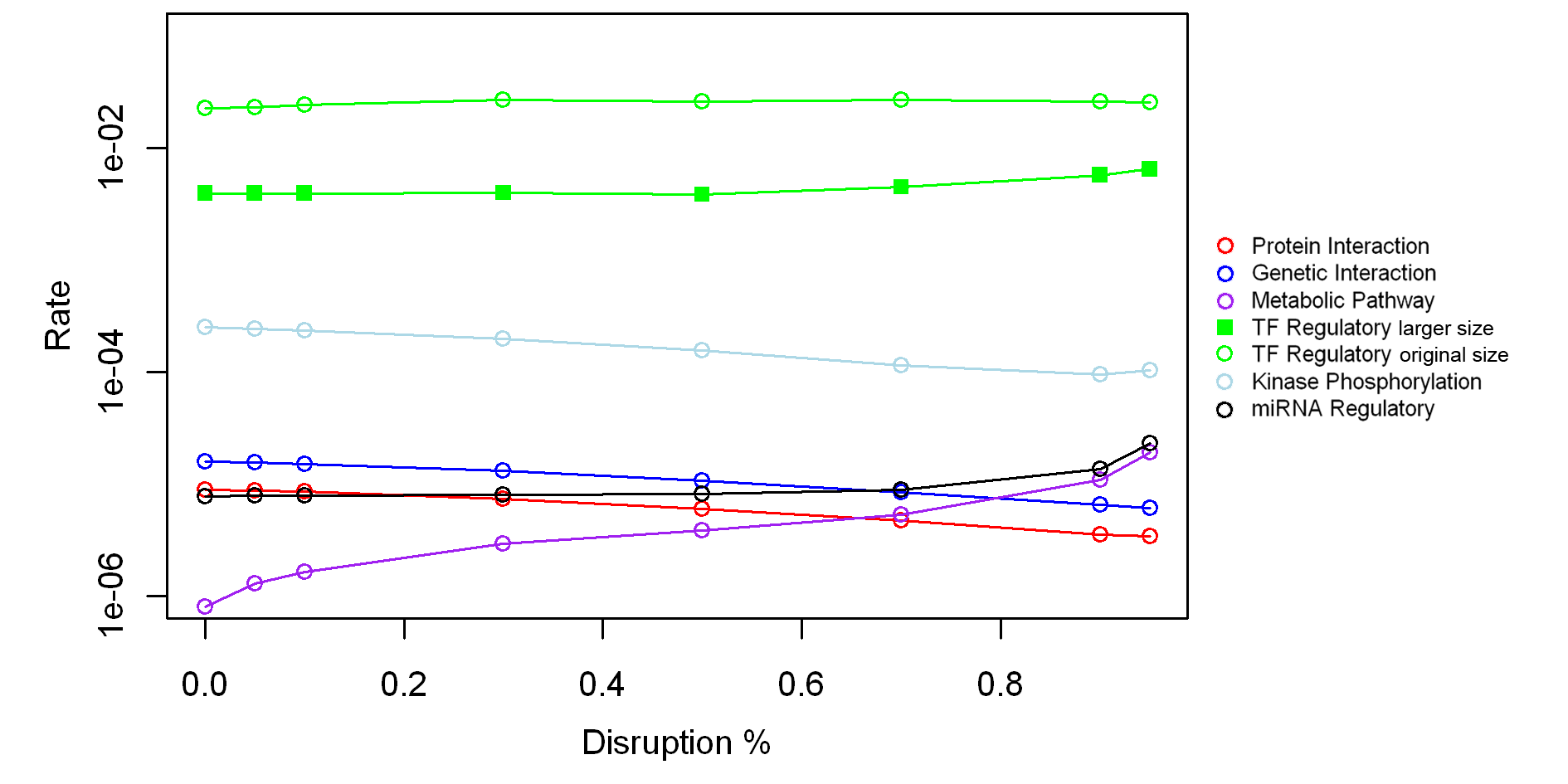

Supplement: Figure S5 — Sensitivity analysis of false positive and false negative rates to rewiring rate. We sampled biological networks in order to test the false positive and false negative rates to rewiring rate calculation. Six biological networks are included here: protein interaction network, genetic interaction network, miRNA-target regulatory network, kinase-substrate phosphorylation network, metabolic pathway network (S. cerevisiae compared to S. pombe) and transcription factor target regulatory network (S. cerevisiae compared to S. bayanus). For each type of network, we randomly delete and add edges from the original network as a simulation of false positives and false negatives, with each a series of percentage disruptions. For transcription factor target regulatory network, we also tested rewiring rate sensitivity to network size by using a larger original network for S. cerevisiae. (0.11 MB TIF) [file pcbi.1001050.s005.tif]

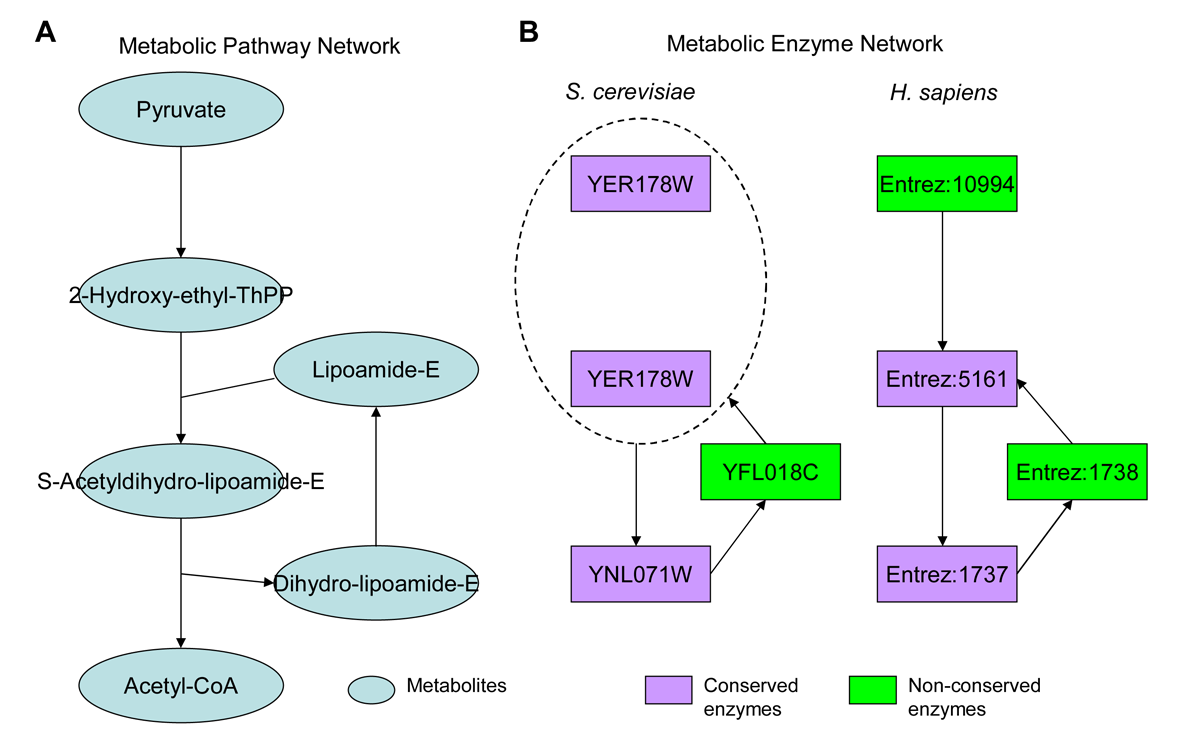

Supplement: Figure S6 — Example rewiring of metabolic pathway network and metabolic enzyme network. (A) The biosynthesis pathway of acetyl-CoA from pyruvate showing metabolites (circles) and reactions (arrows). The pathway is identical in human and yeast. (B) The corresponding metabolic enzyme networks in yeast and human showing enzymes (rectangles) and product-substrate relationships (arrows). Each enzyme corresponds to a reaction in (A). Purple rectangles represent orthologous enzymes from two species, while green rectangles represent non-orthologous enzymes. The dashed circle shows one yeast enzyme coded by YER178W catalyzes two consecutive reactions, but different enzymes catalyze each reaction in human. (0.19 MB TIF) [file pcbi.1001050.s006.tif]

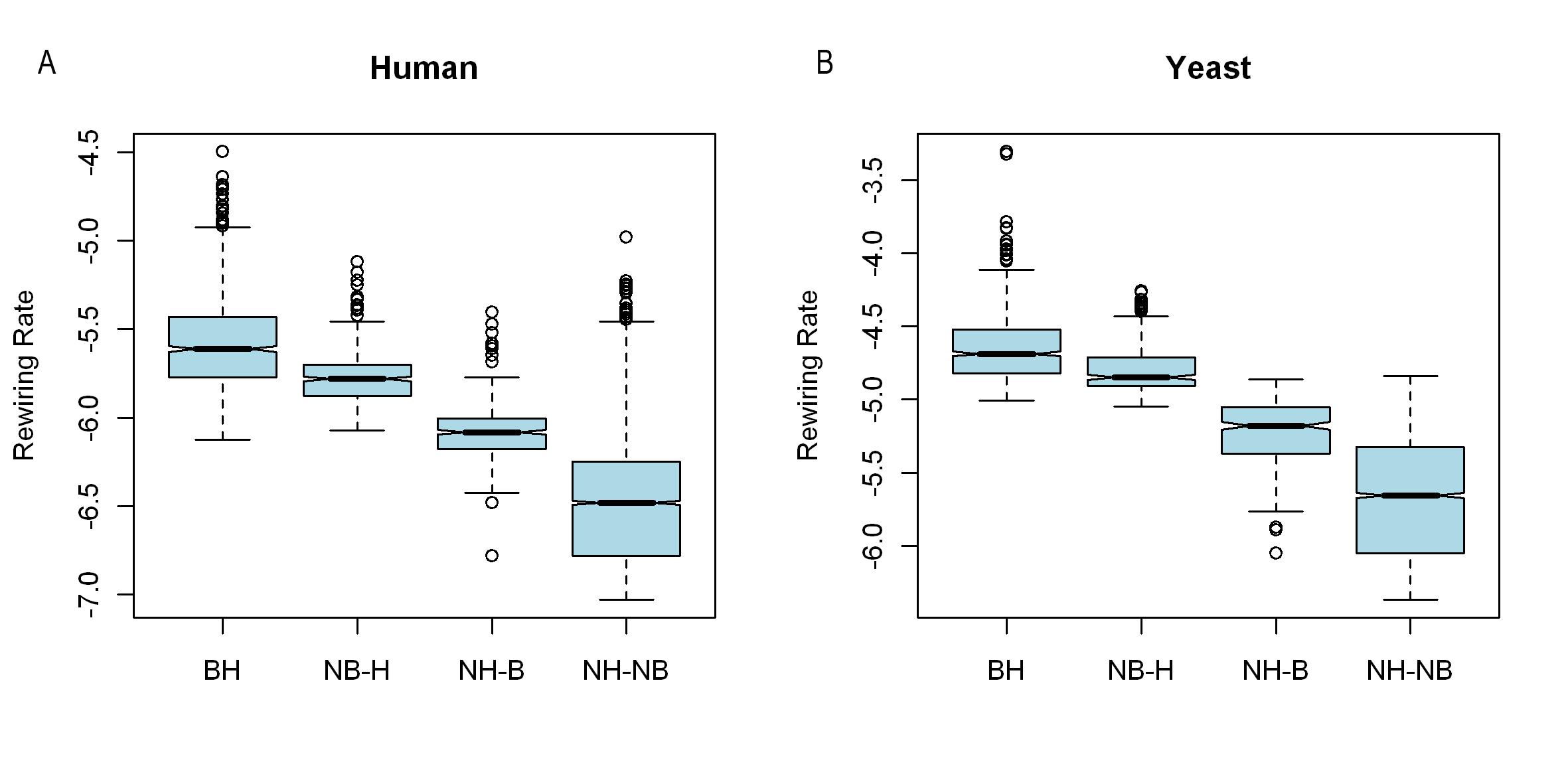

Supplement: Figure S7 — Rewiring rate of hubs and bottlenecks in protein interaction networks. Rewiring rates are calculated for all proteins in (A) human and (B) yeast protein interaction networks. Hubs are defined as top 20% proteins ranked by their degree, and bottlenecks as top 20% ranked by betweenness. Proteins are grouped into 4 categories: Bottleneck hubs (BH), Non-bottleneck hubs (NB-H), Non-hub bottlenecks (NH-B) and Non-hub non-bottlenecks (NH-NB). Either hubs or bottlenecks are found to have faster rewiring rates than NH-NBs (Wilcoxon p-val<e-15). (0.09 MB TIF) [file pcbi.1001050.s007.tif]
