# Supplementary material for: Measuring the Evolutionary Rewiring of Biological Networks
Source: PLoS Comput Biol. 2011 Jan 6;7(1):e1001050. doi: 10.1371/journal.pcbi.1001050 (PMC3017101; doi:10.1371/journal.pcbi.1001050)
Supplement: Table S1 — Estimated divergence times between species pairs. All species pairs used in this study for calculating rewiring rates comparing species networks are listed with estimated divergence time in evolution. The types of networks used for each of these species pairs are also listed. (0.04 MB DOC) [file pcbi.1001050.s008.doc]

Table S1.

| **Species A** | **Species B** | **Estimated Divergence Time (Mys)** | **Networks Used for Rewiring Analysis** |
| --- | --- | --- | --- |
| *S. cerevisiae* | *S. mikatae* | 10 | Metabolic pathway, TF, |
| *S. cerevisiae* | *S. paradoxus* | 10 | Metabolic pathway, |
| *S. cerevisiae* | *S. bayanus* | 20 | Metabolic pathway, TF, |
| *H. sapiens* | *M. mulatta* | 25 | Metabolic pathway, Metabolic enzyme, |
| *C. elegans* | *C. briggsae* | 30 | Metabolic pathway, miRNA |
| *D. melanogaster* | *D. pseudoobscura* | 50 | Metabolic pathway, |
| *H. sapiens* | *M. musculus* | 75 | Metabolic pathway, Metabolic enzyme, miRNA |
| *S. cerevisiae* | *C. glabrata* | 80 | Metabolic pathway, |
| *S. cerevisiae* | *K. lactis* | 150 | Metabolic pathway, TF, |
| *S. cerevisiae* | *D. hansenii* | 270 | Metabolic pathway, |
| *S. cerevisiae* | *C. albicans* | 270 | Metabolic pathway, TF, Phosphorylation, |
| *S. cerevisiae* | *S. pombe* | 420 | PPI, Genetic, Metabolic pathway, Phosphorylation, |
| *H. sapiens* | *D. rerio* | 450 | miRNA |
| *C. elegans* | *D. melanogaster* | 600 | TF |
| *H. sapiens* | *D. melanogaster* | 800 | PPI, Genetic, Metabolic pathway, Metabolic enzyme, miRNA |
| *H. sapiens* | *C. elegans* | 800 | PPI, Genetic, Metabolic pathway, Metabolic enzyme, miRNA |
| *S. cerevisiae* | *D. melanogaster* | 1500 | TF |
| *S. cerevisiae* | *H. sapiens* | 1500 | PPI, Genetic, Metabolic pathway, Metabolic enzyme, Phosphorylation, |
