# Supplementary material for: Measuring the Evolutionary Rewiring of Biological Networks
Source: PLoS Comput Biol. 2011 Jan 6;7(1):e1001050. doi: 10.1371/journal.pcbi.1001050 (PMC3017101; doi:10.1371/journal.pcbi.1001050)
Supplement: Table S2 — Linear regression models of biological network rewiring rate and divergence time. For each type of biological network, rewiring rates (r) from different species pairs are regressed with divergence time (t), both in Log scale. Pearson correlation coefficient is also calculated. (0.04 MB DOC) [file pcbi.1001050.s009.doc]

Table S2.

| **Biological Network** | **Linear Regression Model** | **Correlation Coefficient** |
| --- | --- | --- |
| Transcription factor regulatory network | $\log(\text{r)}=(-1.32\pm0.63)\times\log(t)+(0.22\pm1.43)$ | -0.95 |
| Kinase phosphorylation network | $\log(\text{r)}=(-1.66\pm1.97)\times\log(t)+(0.62\pm5.45)$ | -0.96 |
| miRNA regulatory network | $\log(\text{r)}=(-1.47\pm1.61)\times\log(t)+(-1.24\pm3.68)$ | -0.59 |
| Protein interaction network | $\log(\text{r)}=(-1.64\pm11.83)\times\log(t)+(-0.90\pm34.43)$ | -0.77 |
| Genetic interaction network | $\log(\text{r)}=(-1.09\pm1.59)\times\log(t)+(-1.92\pm4.61)$ | -0.97 |
| Metabolic enzyme network | $\log(\text{r)}=(-0.97\pm0.09)\times\log(t)+(-1.98\pm0.22)$ | -0.54 |
| Metabolic pathway network | $\log(\text{r)}=(-0.70\pm0.24)\times\log(t)+(-4.21\pm0.5)$ | -0.75 |

95% confidence intervals for the fitted parameters are computed for linear models.
