# Supplementary material for: Measuring the Evolutionary Rewiring of Biological Networks
Source: PLoS Comput Biol. 2011 Jan 6;7(1):e1001050. doi: 10.1371/journal.pcbi.1001050 (PMC3017101; doi:10.1371/journal.pcbi.1001050)
Supplement: Table S3 — Detailed rewiring rates for networks and species pairs. Detailed information of rewiring rate results for all networks and species-pairs studied. Numbers of common nodes, gain nodes and loss nodes are provided. Four types of rewired edges (gain edge between common nodes, loss edge between common nodes, gain edge involving gain/loss nodes, loss edge involving gain/loss nodes) are also distinguished for separate rewiring rates. Note for biological networks, rewiring rates are measured by per edge per Mys, while for commonplace networks by per edge per year. (0.17 MB DOC) [file pcbi.1001050.s010.doc]

Table S3.

| **Network Type** | **Species Pair (reference, compared)** | **Divergence (Mys)** | **Shared edges** | **Edge change from Edge Gain** | **Edge change from Edge Loss** | **Edge change from Node Gain** | **Edge change from Node Loss** | **Total possible edges** | **Percentage of rewiring by gene content turnover** | **Total Rate** | **Edge Gain Rate** | **Edge Loss Rate** | **Node Gain Rate** | **Node Loss Rate** | **Common Nodes** | **Gain Nodes** | **Loss Nodes** |
| --- | --- | --- | --- | --- | --- | --- | --- | --- | --- | --- | --- | --- | --- | --- | --- | --- | --- |
| TF | *D. melanogaster, S. cerevisiae* | 1500 | 3 | 80 | 80 | 12733 | 76543 | 1368376 | 0.06535923 | 4.36E-05 | 2.64E-05 | 2.64E-05 | 1.24E-05 | 7.47E-05 | 1011 | 3508 | 10867 |
| TF | *D. melanogaster, C. elegans* | 600 | 0 | 0 | 0 | 33752 | 76626 | 857710 | 0.1286892 | 2.14E-04 | 0 | 0 | 3.27E-04 | 1.86E-04 | 1833 | 6014 | 10045 |
| TF | *S. cerevisiae, C. albicans* | 270 | 54 | NA | NA | 677 | 193 | 924 | NA | 3.50E-03 | NA | NA | 3.40E-03 | 2.90E-03 | 55 | 193 | 677 |
| TF | *S. cerevisiae, K. lactis* | 150 | 95 | NA | NA | 519 | 152 | 766 | NA | 5.80E-03 | NA | NA | 5.60E-03 | 4.10E-03 | 96 | 152 | 519 |
| TF | *S. cerevisiae, S. bayanus* | 20 | 288 | 26 | 53 | 60 | 306 | 986 | 0.82247191 | 2.30E-02 | 3.10E-03 | 6.30E-03 | 2.80E-02 | 3.30E-02 | 213 | 53 | 229 |
| TF | *S. cerevisiae, S. mikatae* | 10 | 328 | 29 | 41 | 70 | 278 | 972 | 0.83253589 | 4.30E-02 | 6.00E-03 | 8.50E-03 | 7.60E-02 | 7.00E-02 | 242 | 46 | 200 |
| PPI | *S. cerevisiae, H. sapiens* | 1500 | 448 | 4189 | 554 | 48668 | 29693 | 48819933 | 0.94292693 | 1.10E-06 | 6.90E-06 | 9.80E-07 | 2.40E-06 | 5.70E-07 | 915 | 4407 | 7460 |
| PPI | *H. sapiens, C. elegans* | 800 | 45 | 289 | 408 | 4250 | 30242 | 38870600 | 0.98019267 | 1.10E-06 | 2.20E-06 | 3.10E-06 | 1.40E-06 | 1.10E-06 | 583 | 2233 | 7792 |
| PPI | *H. sapiens, D. melanogaster* | 800 | 113 | 1044 | 1778 | 21451 | 28804 | 58913366 | 0.94683196 | 1.10E-06 | 1.40E-06 | 2.30E-06 | 1.10E-06 | 1.10E-06 | 1405 | 5641 | 6970 |
| PPI | *S. cerevisiae, S. pombe* | 420 | 1093 | 654 | 4403 | 552 | 48405 | 14596540 | 0.90637612 | 8.80E-06 | 5.80E-06 | 3.90E-05 | 8.50E-06 | 8.10E-06 | 734 | 186 | 4640 |
| Genetic | *S. cerevisiae, H. sapiens* | 1500 | 0 | 2 | 1 | 57851 | 280 | 9666463 | 0.9999484 | 4.00E-06 | 5.80E-06 | 2.90E-06 | 4.00E-06 | 4.50E-06 | 21 | 4366 | 267 |
| Genetic | *H. sapiens, C. elegans* | 800 | 0 | 1 | 0 | 2104 | 281 | 570486 | 0.99958089 | 5.20E-06 | 3.50E-05 | 0 | 5.00E-06 | 8.40E-06 | 8 | 1020 | 280 |
| Genetic | *H. sapiens, D. melanogaster* | 800 | 0 | 18 | 6 | 5206 | 275 | 520765 | 0.99564033 | 1.30E-05 | 4.00E-05 | 1.30E-05 | 1.40E-05 | 8.40E-06 | 33 | 946 | 255 |
| Genetic | *S. cerevisiae, S. pombe* | 420 | 1261 | 5925 | 10191 | 3287 | 46441 | 10007207 | 0.75523966 | 1.60E-05 | 3.80E-05 | 6.60E-05 | 3.30E-05 | 1.20E-05 | 858 | 242 | 3563 |
| Phosphorylation | *H. sapiens, S. cerevisiae* | 1500 | 0 | 87 | 114 | 3981 | 27806 | 933247 | 0.99371639 | 2.20E-05 | 2.10E-05 | 2.70E-05 | 2.40E-05 | 2.30E-05 | 123 | 1244 | 2413 |
| Phosphorylation | *S. cerevisiae, S. pombe* | 420 | 226 | 299 | 277 | 1817 | 3053 | 58391 | 0.8942343 | 2.20E-04 | 1.30E-04 | 1.20E-04 | 2.30E-04 | 2.10E-04 | 154 | 325 | 551 |
| Phosphorylation | *S. cerevisiae, C. albicans* | 270 | 385 | 474 | 383 | 4260 | 2788 | 84823 | 0.8915876 | 3.50E-04 | 2.20E-04 | 1.80E-04 | 3.50E-04 | 3.20E-04 | 192 | 737 | 513 |
| miRNA | *H. sapiens, C. elegans* | 800 | 3 | 2 | 0 | 122 | 5679 | 1785268 | 0.99965535 | 4.10E-06 | 8.90E-05 | 0 | 3.30E-05 | 4.00E-06 | 11 | 133 | 4199 |
| miRNA | *H. sapiens, D. melanogaster* | 800 | 20 | 9 | 1 | 99 | 5661 | 1784155 | 0.9982669 | 4.00E-06 | 2.80E-05 | 3.10E-06 | 3.50E-05 | 4.00E-06 | 43 | 104 | 4167 |
| miRNA | *H. sapiens, D. rerio* | 450 | 300 | 198 | 168 | 914 | 5214 | 1875715 | 0.94364028 | 7.70E-06 | 1.10E-05 | 9.70E-06 | 2.10E-05 | 6.70E-06 | 468 | 742 | 3742 |
| miRNA | *H. sapiens, M. musculus* | 75 | 2138 | 410 | 477 | 3178 | 3067 | 2850669 | 0.87563096 | 3.30E-05 | 7.80E-06 | 9.10E-06 | 4.00E-05 | 3.80E-05 | 1987 | 2275 | 2214 |
| miRNA | *C. elegans, C. briggsae* | 30 | 12 | 1 | 0 | 44 | 115 | 6212 | 0.99375 | 8.60E-04 | 1.20E-04 | 0 | 9.40E-04 | 8.80E-04 | 35 | 51 | 109 |
| Metabolic Pathway | *H. sapiens, S. cerevisiae* | 1500 | 1099 | 64 | 158 | 208 | 652 | 1940402 | 0.7948244 | 3.70E-07 | 7.10E-08 | 1.70E-07 | 5.60E-07 | 4.00E-07 | 778 | 145 | 524 |
| Metabolic Pathway | *H. sapiens, C. elegans* | 800 | 1191 | 13 | 192 | 47 | 520 | 1783038 | 0.73445596 | 5.40E-07 | 2.00E-08 | 2.90E-07 | 6.60E-07 | 7.40E-07 | 905 | 48 | 397 |
| Metabolic Pathway | *H. sapiens, D. melanogaster* | 800 | 1400 | 43 | 160 | 102 | 340 | 1922026 | 0.68527132 | 4.20E-07 | 5.20E-08 | 2.00E-07 | 5.60E-07 | 6.40E-07 | 1013 | 107 | 289 |
| Metabolic Pathway | *S. cerevisiae, S. pombe* | 420 | 1133 | 19 | 87 | 78 | 154 | 1031728 | 0.68639053 | 7.80E-07 | 7.50E-08 | 3.50E-07 | 1.00E-06 | 1.50E-06 | 775 | 109 | 148 |
| Metabolic Pathway | *S. cerevisiae, C. albicans* | 270 | 463 | 18 | 179 | 19 | 743 | 869746 | 0.79457769 | 4.10E-06 | 3.20E-07 | 3.20E-06 | 3.80E-06 | 4.30E-06 | 459 | 20 | 464 |
| Metabolic Pathway | *S. cerevisiae, D. hansenii* | 270 | 1196 | 19 | 80 | 196 | 97 | 1323634 | 0.74744898 | 1.10E-06 | 9.80E-08 | 4.10E-07 | 1.50E-06 | 2.70E-06 | 847 | 244 | 78 |
| Metabolic Pathway | *S. cerevisiae, K. lactis* | 150 | 1146 | 7 | 100 | 102 | 128 | 1097612 | 0.68249258 | 2.10E-06 | 6.90E-08 | 9.80E-07 | 2.80E-06 | 5.00E-06 | 825 | 138 | 98 |
| Metabolic Pathway | *S. cerevisiae, C. glabrata* | 80 | 1204 | 10 | 36 | 56 | 133 | 955560 | 0.80425532 | 3.10E-06 | 1.80E-07 | 6.60E-07 | 6.70E-06 | 9.90E-06 | 827 | 61 | 96 |
| Metabolic Pathway | *H. sapiens, M. musculus* | 75 | 1831 | 11 | 24 | 17 | 45 | 1744282 | 0.63917526 | 7.40E-07 | 9.40E-08 | 2.00E-07 | 4.50E-06 | 4.50E-06 | 1250 | 20 | 52 |
| Metabolic Pathway | *D. melanogaster, D. pseudoobscura* | 50 | 1199 | 24 | 109 | 42 | 238 | 1336356 | 0.6779661 | 6.20E-06 | 5.40E-07 | 2.40E-06 | 1.00E-05 | 1.30E-05 | 945 | 43 | 175 |
| Metabolic Pathway | *C. elegans, C. briggsae* | 30 | 1196 | 73 | 25 | 190 | 31 | 1282064 | 0.69278997 | 8.30E-06 | 2.80E-06 | 9.70E-07 | 1.70E-05 | 2.10E-05 | 927 | 184 | 26 |
| Metabolic Pathway | *H. sapiens, M. mulatta* | 25 | 1706 | 12 | 90 | 10 | 106 | 1728384 | 0.53211009 | 5.00E-06 | 3.20E-07 | 2.40E-06 | 1.20E-05 | 2.20E-05 | 1225 | 14 | 77 |
| Metabolic Pathway | *S. cerevisiae, S. bayanus* | 20 | 1299 | 23 | 40 | 48 | 32 | 963026 | 0.55944056 | 7.40E-06 | 1.40E-06 | 2.50E-06 | 2.10E-05 | 4.60E-05 | 904 | 60 | 19 |
| Metabolic Pathway | *S. cerevisiae, S. mikatae* | 10 | 1303 | 16 | 36 | 61 | 32 | 994114 | 0.64137931 | 1.50E-05 | 2.00E-06 | 4.40E-06 | 4.30E-05 | 9.20E-05 | 904 | 76 | 19 |
| Metabolic Pathway | *S. cerevisiae, S. paradoxus* | 10 | 1305 | 6 | 32 | 39 | 34 | 947466 | 0.65765766 | 1.20E-05 | 7.40E-07 | 3.90E-06 | 4.00E-05 | 8.90E-05 | 902 | 52 | 21 |
| Metabolic Enzyme | *H. sapiens, S. cerevisiae* | 1500 | 503 | 54 | 52 | 5301 | 15147 | 1634182 | 0.99484285 | 8.40E-06 | 1.10E-06 | 1.10E-06 | 9.10E-06 | 8.30E-06 | 182 | 467 | 935 |
| Metabolic Enzyme | *H. sapiens, C. elegans* | 800 | 506 | 44 | 12 | 4431 | 15185 | 1490166 | 0.99715331 | 1.70E-05 | 1.70E-06 | 4.80E-07 | 2.30E-05 | 1.60E-05 | 178 | 347 | 939 |
| Metabolic Enzyme | *H. sapiens, D. melanogaster* | 800 | 586 | 132 | 38 | 21080 | 15064 | 2644584 | 0.99531861 | 1.70E-05 | 3.30E-06 | 9.40E-07 | 1.90E-05 | 1.60E-05 | 225 | 979 | 892 |
| Metabolic Enzyme | *H. sapiens, M. musculus* | 75 | 2699 | 116 | 104 | 11863 | 12744 | 2146602 | 0.99113868 | 1.50E-04 | 6.10E-06 | 5.40E-06 | 1.80E-04 | 1.80E-04 | 505 | 570 | 612 |
| Metabolic Enzyme | *H. sapiens, M. mulatta* | 25 | 1263 | 16 | 0 | 6947 | 14368 | 1762542 | 0.99924992 | 4.80E-04 | 4.80E-06 | 0 | 5.40E-04 | 5.20E-04 | 365 | 441 | 752 |
| Linux | *V4, V15* | 2 yrs | 11072 | 877 | 3189 | 11981 | 2696 | 55228321 | 0.78306568 | 1.70E-04 | 2.50E-05 | 9.00E-05 | 1.90E-04 | 2.20E-04 | 8498 | 5585 | 1334 |
| Linux | *V4, V27* | 4.5 yrs | 7111 | 1156 | 4213 | 25451 | 5633 | 107762336 | 0.85271445 | 7.50E-05 | 2.00E-05 | 7.20E-05 | 6.70E-05 | 1.10E-04 | 7286 | 12506 | 2546 |
| Family | *1983, 2009* | 26 yrs | 19 | 0 | 1 | 25 | 19 | 1821 | 0.97777778 | 9.50E-04 | 0 | 2.20E-04 | 8.30E-04 | 1.50E-03 | 19 | 33 | 18 |
| Co-authorship | *2006, 2009* | 3 yrs | 8 | 0 | 1 | 38 | 38 | 445 | 0.98701299 | 5.80E-02 | 0 | 1.60E-02 | 5.00E-02 | 7.50E-02 | 7 | 17 | 13 |
