# Supplementary material for: Measuring the Evolutionary Rewiring of Biological Networks
Source: PLoS Comput Biol. 2011 Jan 6;7(1):e1001050. doi: 10.1371/journal.pcbi.1001050 (PMC3017101; doi:10.1371/journal.pcbi.1001050)
Supplement: Table S4 — Simulation of network size, false positives, and false negatives to rewiring rate. Based on two simulated scale-free networks, sub-networks are sampled to mimic the fact that data of many biological networks used in this study are not complete, such as the fungi TF regulatory networks. Extra random rewiring by adding and removing edges and nodes is performed to mimic the false positives and negatives in the current network data. Percentage of network rewiring is then calculated to assess the effects of those perturbations. (0.05 MB DOC) [file pcbi.1001050.s011.doc]

Table S4.

| **Sub-sampling fraction** | **Rewiring percentage** | **Rewired edges** | **Shared edges** | **Total possible edge changes** | **Shared nodes** | **Unique node in network A** | **Unique node in network B** |
| --- | --- | --- | --- | --- | --- | --- | --- |
| 100% | 4.3e-4±7e-6 | 11660±130 | 6054±156 | 2.7e7±3e5 | 6867±24 | 505±52 | 1±0 |
| 95% | 4.5e-4±6e-6 | 11690±117 | 5576±146 | 2.6e7±3e5 | 6494±38 | 594±66 | 101±7 |
| 90% | 4.8e-4±5e-6 | 11596±134 | 5129±112 | 2.4e7±2e5 | 6126±23 | 651±39 | 190±15 |
| 70% | 5.8e-4±7e-6 | 10911±136 | 3377±35 | 1.9e7±9e4 | 4898±34 | 742±20 | 566±21 |
| 50% | 6.2e-4±7e-6 | 9577±130 | 1875±62 | 1.6e7±7e4 | 3855±25 | 1013±22 | 864±15 |
| 30% | 6.8e-4±6e-6 | 7187±64 | 767±19 | 1.0e7±9e4 | 2589±24 | 1227±27 | 1017±10 |
| 10% | 9.4e-4±1e-5 | 3136±48 | 95±5 | 3.3e6±3e4 | 1030±15 | 1008±18 | 857±16 |
| 5% | 1.3e-3±2e-5 | 1801±14 | 29±2 | 1.4e6±3e4 | 545±16 | 756±27 | 642±11 |
| 3% | 1.7e-3±4e-5 | 1167±29 | 12±2 | 6.7e5±3e4 | 306±15 | 580±27 | 497±14 |
| 1% | 3.9e-3±8e-5 | 431±21 | 2±0.5 | 1.1e5±7e3 | 75±4 | 290±14 | 236±11 |

For each sub-sampling fraction, we performed 10 simulations and calculated 95% confidence intervals for resulting numbers.
