# Supplementary material for: Measuring the Evolutionary Rewiring of Biological Networks
Source: PLoS Comput Biol. 2011 Jan 6;7(1):e1001050. doi: 10.1371/journal.pcbi.1001050 (PMC3017101; doi:10.1371/journal.pcbi.1001050)
Supplement: Table S5 — Simulation analysis of the effect of novel miRNAs to miRNA regulatory network. Based on current miRNA regulatory networks for human and mouse, simulated novel miRNAs are added to both networks with their target randomly sampled, while maintaining the power-law distribution of target number distribution. Statistics are calculated comparing the simulated networks. (0.04 MB DOC) [file pcbi.1001050.s012.doc]

Table S5.

| **miRs added** | **Rewiring rate** | **Rewired edges** | **Shared edges** | **Total possible edge changes** | **Shared miRs** | **Unique miRs in network A** | **Unique miRs in network B** |
| --- | --- | --- | --- | --- | --- | --- | --- |
| 0 | 3.3e-5 | 7132 | 2138 | 2.9e6 | 459 | 18 | 9 |
| 50 | 6.9e-5±1e-5 | 16800±3174 | 2138 | 3.2e6 | 459 | 68 | 59 |
| 100 | 1.7e-4±2e-5 | 44640±6281 | 2138 | 3.6e6 | 459 | 118 | 109 |
| 200 | 3.1e-4±4e-5 | 102110±12889 | 2138 | 4.4e6 | 459 | 218 | 209 |
| 400 | 4.9e-4±6e-5 | 215559±25995 | 2138 | 5.9e6 | 459 | 418 | 409 |
| 600 | 7.2e-4±7e-5 | 396853±38672 | 2138 | 7.4e6 | 459 | 618 | 609 |
| 800 | 9.7e-4±8e-5 | 647465±50143 | 2138 | 8.9e6 | 459 | 818 | 809 |

For each number of added miRNAs, we performed 10 simulations by assigning different targets to the added miRs, and calculated 95% confidence intervals for resulting numbers.
